# Bioenergetic adaptations of small intestinal epithelial cells reduce cell differentiation enhancing intestinal permeability in obese mice

**DOI:** 10.1016/j.molmet.2025.102098

**Published:** 2025-01-13

**Authors:** Thomas Guerbette, Vincent Ciesielski, Manon Brien, Daniel Catheline, Roselyne Viel, Mégane Bostoën, Jean-Baptiste Perrin, Agnès Burel, Régis Janvier, Vincent Rioux, Annaïg Lan, Gaëlle Boudry

**Affiliations:** 1 Institut Numecan, INRAE, INSERM, Univ Rennes, Rennes, France; 2 Institut Agro Rennes Angers, Rennes, France; 3 Univ Rennes, CNRS, Inserm, Biosit UAR 3480 US_S 018, France-BioImaging (ANR-10-INBS-04), plateforme H2P2, Rennes, France; 4 Plateforme MRic, UMS 3480 BIOSIT, Rennes, France; 5 Université Paris-Saclay, AgroParisTech, INRAE, UMR PNCA, 91120, Palaiseau, France

**Keywords:** High fat diet, Intestine, Lipid metabolism, Mitochondria, Obesity

## Abstract

****Objective**:**

Obesity and overweight are associated with low-grade inflammation induced by adipose tissue expansion and perpetuated by altered intestinal homeostasis, including increased epithelial permeability. Intestinal epithelium functions are supported by intestinal epithelial cells (IEC) mitochondria function. However, diet-induced obesity (DIO) may impair mitochondrial activity of IEC and consequently, intestinal homeostasis. The aim of the project was to determine whether DIO alters the mitochondrial function of IEC, and what are the consequences on intestinal homeostasis.

**Methods:**

C57Bl/6J mice were fed a control diet for 22 weeks or a high fat diet (58 kcal% fat). Bioenergetic adaptations of IEC were evaluated on isolated crypts and villi from mouse jejunum. To determine the link between mitochondrial function and alterations of intestinal homeostasis in response to lipid overload, we used the jejunal epithelial cell line IPEC-J2 *in vitro* and mouse jejunum organoids.

**Results:**

Here, we report that DIO in mice induced lipid metabolism adaptations favoring lipid storage in IEC together with reduced number, altered dynamics and diminished oxidative phosphorylation activity of IEC mitochondria. Using the IPEC-J2 cell line, we showed that IEC lipid metabolism and oxidative stress machinery adaptations preceded mitochondrial bioenergetic ones. Moreover, we unraveled the intricate link between IEC energetic status and proliferation / differentiation balance since enhancing mitochondrial function with the AMPK activator AICAR in jejunal organoids reduced proliferation and initiated IEC differentiation and conversely. We confirmed that the reduced IEC mitochondrial function observed in DIO mice was associated with increased proliferation and reduced differentiation, promoting expression of the permissive *Cldn2* in the jejunal epithelium of DIO mice.

**Conclusions:**

Our study provides new insights into metabolic adaptations of IEC in obesity by revealing that excess lipid intake diminishes mitochondrial number in IEC, reducing IEC differentiation that contribute to increased epithelial permeability.

## Highlights

• High fat diet-induced lipid metabolism adaptations favor fat storage in enterocytes.
• These metabolic adaptations are accompanied by a reduction in mitochondrial number.
• Reduced mitochondrial number provokes a decrease in mitochondrial activity.
• Low mitochondrial activity favors immature phenotype of enterocytes in obesity.
• Immature intestinal phenotype participates in the increase of intestinal permeability in obesity.

## Graphical abstract

**Figure:**
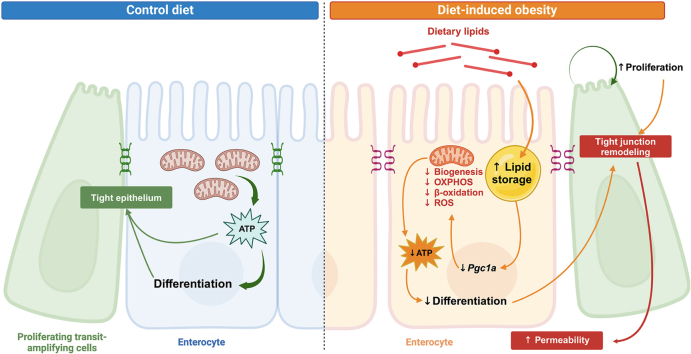

## 1 Introduction

Obesity and associated complications, such as diabetes and cardiovascular diseases, causes the death of 4 million people each year [1]. Systemic low-grade inflammation responsible of these obesity-associated complications is induced by adipose tissue expansion [2,3] and perpetuated by altered intestinal homeostasis [4]. Animal models of diet-induced obesity (DIO) [5,6] and obese humans [7] display increased small intestine permeability, resulting from tight junction (TJ) protein remodeling [8]. Several studies established that high fat diet (HFD) consumption enhances stemness of mouse small intestine progenitors [[9], [10], [11]], resulting in increased number of intestinal stem cells (ISC) and higher proliferation of intestinal epithelial cells (IEC). The fine-tuned balance between IEC proliferation and differentiation plays a key role in the regulation of intestinal epithelium permeability. Indeed, enhancement of IEC proliferation by stress is associated with heightened intestinal permeability in rat submitted to psychological stress [12]. These data thus suggest that TJ protein remodeling could result from HFD-induced change in IEC proliferation/differentiation balance favoring increased permeability. Differentiated IEC are characterized by high mitochondrial content, regulated by a high expression of PGC1α, a key factor involved in mitochondrial biogenesis, compared to proliferating cells at the bottom of intestinal crypts [13]. Parallel phenotypic and metabolic transitions occur during cellular differentiation [14], from high glycolytic activities in ISC towards enhanced oxidative metabolism and dependency of IEC [15]. Yet, whether enhanced oxidative phosphorylation (OXPHOS) activity is simply the result of increased energy need for nutrient absorption and TJ maintenance in fully differentiated IEC or the differentiation process itself is driven by enhanced mitochondrial function, is still not established. One study demonstrated in small intestine organoids that OXPHOS activity was required when inducing crypt formation by modulating the culture medium composition [16].

Until now, only mitochondrial function of colonic epithelial cells in DIO models has been investigated [[17], [18], [19], [20]]. Bioenergetic adaptations of IEC under HFD remain poorly described so far in the small intestine. Yet, it has been reported that HFD increased fatty acid β-oxidation (FAO) of mouse ISC [11] and in isolated mitochondria from mouse small intestine mucosa after 2 weeks of HFD [21]. Besides, the expression of *Pgc1a* is decreased by half in IEC of DIO mice [22] indicating probable alterations of mitochondrial biogenesis. Noteworthy, *Pgc1a* expression is modulated by the energetic sensor AMP-activated protein kinase (AMPK), whose activation was shown to be reduced in a context of obesity and type 2 diabetes [23]. Nevertheless, strong evidence of IEC mitochondrial dysfunction induced by HFD in the small intestine are lacking so far.

Interestingly, excess of saturated fatty acid has been shown to impair mitochondrial function in several tissues [[24], [25], [26]], through mechanisms linked to increased FAO, OXPHOS uncoupling and associated oxidative stress [[27], [28], [29], [30]]. In IEC, lipid metabolism adaptation to HFD is still unclear. Several studies indicated that HFD promotes a rapid metabolic shift toward lipid absorption and catabolism in IEC [21,31,32], likely to counteract excessive lipid storage as observed in hepatocytes during nonalcoholic fatty liver disease [33]. However, the presence of cytosolic lipid droplets in IEC has also been reported in humans [34,35] and in several animal models of DIO [36,37], likely to favor the storage of fatty acids that are not oxidized or exported from IEC. Besides, it has been recently reported that mitochondrial function of IEC plays a major role in dietary lipid transport and notably by allowing the export of chylomicrons [38].

We hypothesized that excess dietary fatty acid leads to alterations of lipid metabolism in IEC with concomitant mitochondrial adaptations, generating perturbations of the small intestinal epithelium renewal and remodeling of TJ, enhancing small intestinal permeability. We thus thoroughly characterized mitochondrial function and lipid metabolism in IEC in relation with IEC proliferation/differentiation balance and epithelial permeability in a mouse model of DIO. To decipher the sequence of events, we also used an *in vitro* model of jejunal epithelial cells with a cell metabolism close to *in vivo* IEC as well as mouse jejunal organoids.

## 2 Material and methods

### 2.1 Model of diet-induced obesity in mice

Animal procedures were performed in accordance with French law and approved by the *Comité Rennais d’Ethique en Expérimentation Animale* and by the *Ministère de l’Enseignement Supérieur et de la Recherche* (APAFIS#22076–2019091911225127 v4). Male C57BL/6J mice (Janvier, France) were acclimated for one week to housing conditions and fed chow diet (16% protein; Envigo, United States) and tap water *ad libitum*. Mice were housed by group of three to five per cage in an environment maintained at 21 °C with a 12:12h light–dark cycle. After acclimation, half of the animals (CTRL group) received *ad libitum* either a control diet (D12328, 10% kcal fat Research Diets, *n* = 17) and tap water or an obesogenic diet (D12331, 58% kcal fat, *n* = 15) and drinking water enriched with carbohydrates (45% sucrose and 55% fructose, 42 g/L) to induce obesity (DIO group). After 22 weeks, animals were anesthetized with isoflurane and blood samples were collected by intra-cardiac puncture. After cervical dislocation, fat pads and liver were weighed and stored at −80 °C or fixed in 4% buffered formaldehyde for histology. Eight centimeters from proximal jejunum were sampled 5 cm after the pylorus, flushed with cold Hanks' Balanced Salt Solution (HBSS, Gibco, 14170112) and stored in cold HBSS for immediate IEC isolation. The following centimeter of jejunum was maintained in Krebs buffer for immediate permeability assay in Ussing chamber while another centimeter was fixed in 4% buffered formaldehyde for histology. One centimeter dissected in 5 mm pieces of jejunum was rinsed with 0.15 M sodium cacodylate buffer and fixed by adding 2.5% glutaraldehyde for 1 h for further electron microscopy. Blood samples were left on ice for 2 h and then centrifugated at 240×*g* for 10 min. Serum was aliquoted and stored at −80 °C. Serum alanine aminotransferase (ALAT), triglycerides and cholesterol assays were performed at the Biochemistry Department of Rennes University Hospital.

### 2.2 Jejunal epithelial cell isolation

Jejunal villus and crypt isolation protocol was adapted from [39]. Five square-millimeter pieces were cut and incubated into 10 mL ice-cold 5 mM ethylenediaminetetraacetic acid - HBSS at 4 °C in a benchtop roller. After the incubation and washes, samples were vigorously shaken for 30 s in 30 mL HBSS. Isolated villi and crypts were centrifugated at 200×*g* for 5 min and resuspended into 6 mL of Advanced DMEM/F-12 (ADF; Gibco, 12634010) supplemented with 1% penicillin/streptomycin (P/S Thermo Fisher Scientific, 15140122), and 1% HEPES (Gibco, 15630106). Isolated IEC were immediately used for metabolic analyses (bioenergetic analysis, FAO), fatty acid quantification and reactive oxygen species (ROS) detection assays while the remaining cells were centrifugated at 200×*g* for 5 min and either resuspended in lysis buffer RA1 (Macherey–Nagel) for RNA extraction, or in RIPA for further protein extraction, and stored at −80 °C. The IEC yield per isolation was determined by quantifying the total amount of proteins obtained after isolation and was not significantly different between groups (Supplementary Fig. 1).

### 2.3 Culture of mouse small intestine organoids

Isolated crypts and villi from CTRL mice were filtered through a 70 μm filter (VWR International, 2572267) to retain crypts. After centrifugation of the suspension at 200×*g* for 5 min, 250 crypts were seeded in 25 μL Matrigel (Corning, 354230). The plate was placed in the incubator at 37 °C and 5% CO_2_ for 10 min to allow the Matrigel to solidify. The proliferative medium was added to well and was composed of 45% ADF supplemented with P/S (2%), HEPES (1%), l-Glutamine (1%; Thermo Fisher Scientific, A2916801), fetal bovine serum (FBS; HyClone, SH30066.03), 45% conditioned medium of L-WRN cells, grown in DMEM- GlutaMAX (Thermo Fisher Scientific, 31966-021) with FBS (10%) and 8 nM epidermal growth factor (EGF; Sigma, E9644) and 10% of complete Intesticult (50% of IntestiCult OGM Human Basal Medium (Stemcell, 100–0190), 50% of Organoid Supplement (Stemcell, 100–0191)). This medium was supplemented with 10 mM Y-27632 (Sigma, Y0503) for the first 24 h. Organoids were subcultured once a week at 1:4 (well:well), using successive pipetting of gentle cell dissociation reagent (Stemcell, 07174) onto the Matrigel to break it up and collect organoids. Differentiation was induced for 48 h after 3 days of culture by diluting at 1:10 (vol:vol) the proliferative medium in ADF medium supplemented with P/S, HEPES, l-Glutamine and FBS in the same proportions as mentioned above. 5-Aminoimidazole-4-carboxamide ribonucleoside (AICAR; Sigma, A9978) was resuspended into sterile water at 100 mM. AICAR treatment was performed by adding 2 mM AICAR for 24 h in the proliferative medium after 4 days of organoid proliferation. To evaluate the effect of AICAR treatment on organoid bioenergetic, equivalent amount of organoids were seeded onto a Seahorse plate, as indicated by similar Hoechst intensity between conditions (Supplementary Fig. 2).

### 2.4 IPEC-J2 cell line

IPEC-J2 were maintained in DMEM supplemented with 10% FBS, 1% P/S, in a humidified atmosphere of 5% CO_2_ at 37 °C. Cells were passed once a week and seeded at a density of 100,000 cells/cm^2^. Cells were treated once they reached confluence with an equimolar mix of C12:0 (Sigma, L4250), C14:0 (Sigma, M3128), C16:0 (Sigma, P5585) and C18:0 (Sigma, S4751) at 250 μM each and diluted in dimethyl sulfoxide (DMSO) to a final concentration of 0.6% in culture media.

All methods used in this study are presented in Supplementary material.

## 3 Results

### 3.1 DIO mice display increased adiposity, hepatic steatosis and altered glucose metabolism

Male C57BL/6J were fed a CTRL diet (10% kcal from fat) or a high-fat diet (58% kcal from fat) supplemented with sugars (42 g/L) in drinking water for 22 weeks to induce obesity. DIO mice displayed increased body weight (Supplementary Fig. 3A), abdominal, peritoneal and epidydimal fat pad relative weights (Supplementary Fig. 3.B), hepatic steatosis (Supplementary Fig. 3.C, D) and serum ALAT concentration (Supplementary Fig. 3.E) indicating hepatic cytolysis, compared to CTRL mice. DIO mice were also characterized by enhanced fasting glycemia, a loss of glucose homeostasis compared to CTRL mice (Supplementary Fig. 3.F) and increased concentration of circulating triglycerides and cholesterol (Supplementary Fig. 3.G).

### 3.2 DIO reduces IEC mitochondrial number and function

**Figure 1:**
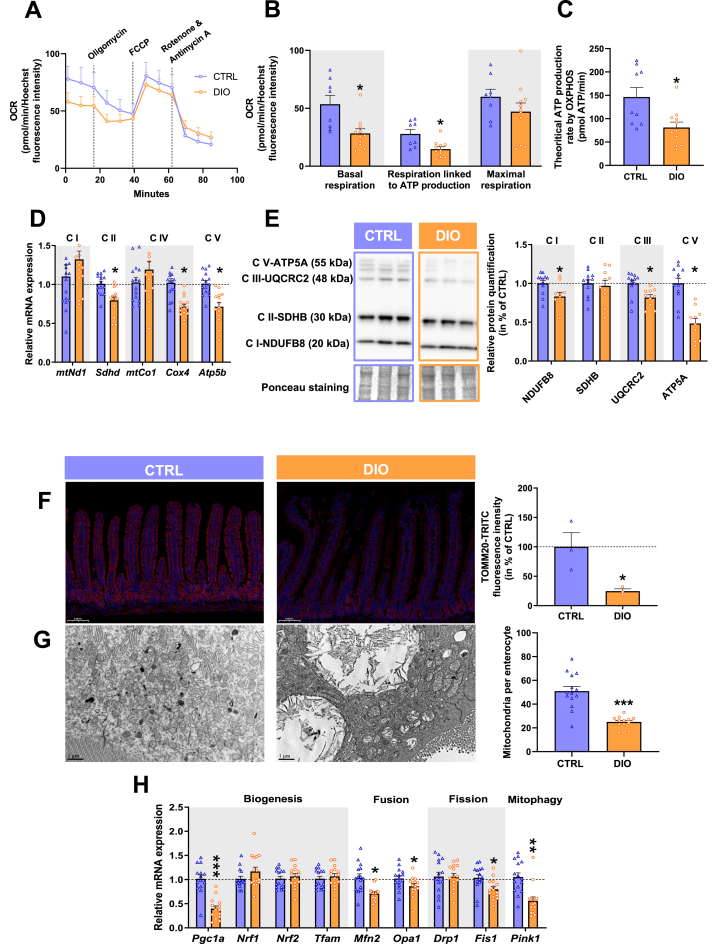
**Diet-induced obesity impairs oxidative phosphorylation capacity of intestinal epithelial cell through reduction of mitochondrial number. A.** Oxygen consumption rate (OCR) of isolated IEC measured after sequential injection of oligomycin, FCCP and rotenone and antimycin A (*N* = 8 CTRL; *N* = 11 DIO). **B.** Mitochondrial basal respiration, respiration linked to ATP production and maximal respiration of isolated intestinal epithelial cells (IEC) calculated from the OCR profiles (*N* = 9 CTRL; *N* = 11 DIO). **C.** Evaluation of theoretical ATP production rate from oxidative phosphorylation (OXPHOS) of IEC (*N* = 9 CTRL; *N* = 11 DIO). **D.** mRNA relative expression of electron transport chain (ETC) subunits. Values are presented as fold change relative to CTRL calculated by the 2^−ΔΔCt^ method (*N* = 15 CTRL; *N* = 15 DIO). **E.** Western blot and semi-quantitative protein expression of subunits from ETC of IEC normalized by Ponceau staining. Semi-quantitative results are expressed as percentage of CTRL means (*N* = 12 CTRL; *N* = 10 DIO). **F.** TOMM20 immunostaining (red) in jejunal mucosa counterstained with DAPI (blue) and measurement of TOMM20 coupled fluorescence intensity normalized by surface area. Data are expressed in percentage of CTRL (*N* = 3 CTRL; *N* = 3 DIO). **G.** Transmission electron images of mitochondria from IEC from jejunal mucosa and mitochondria number per IEC (*N* = 14 cells from 3 CTRL mice; *N* = 12 cells from 3 DIO mice). **H.** mRNA relative expression of genes involved in mitochondrial dynamics in IEC. Values are presented as fold change relative to CTRL calculated by the 2^−ΔΔCt^ method (*N* = 15 CTRL; *N* = 15 DIO). Results are means ± SEM. Significant results are represented as ∗*P* < 0.05, ∗∗*P* < 0.01, ∗∗∗*P* < 0.001. Abreviation: mt = mitochondria. (For interpretation of the references to color in this figure legend, the reader is referred to the Web version of this article.)

IEC were isolated from the proximal jejunum to analyse their mitochondrial function using the Seahorse analyzer (Figure 1.A). Intestinal epithelial cells from DIO mice displayed 50% lower basal mitochondrial respiration and respiration linked to ATP production than CTRL mice (Figure 1.B). Other bioenergetic parameters, remained statistically unchanged (Figure 1.A, B; Supplementary Fig. 4), although maximal respiration appeared reduced in most of the DIO mice, except one. Nevertheless, basal theoretical ATP production rate from OXPHOS, calculated from oxygen consumption rate (OCR), extracellular acidification rate (ECAR) and proton efflux rates (PER) data, was reduced by half in IEC from DIO compared to CTRL mice (Figure 1.C). Taken together, bioenergetic analysis revealed a diminished capacity of IEC mitochondria to produce ATP in DIO (Figure 1.B,C). In line with the lower basal respiration and OXPHOS-derived ATP production of IEC mitochondria in DIO mice, mRNA relative expressions of the electron transport chain (ETC) subunits were 20%–30% lower in IEC from DIO mice compared to CTRL ones (Figure 1.D). Western blot confirmed the reduced protein expression of several subunits of the ETC in IEC from DIO mice (Figure 1.E). Finally, we evaluated mitochondrial mass, first by immunohistochemistry targeting the outer mitochondrial membrane transporter TOMM20, whose intensity was decreased by 75% in the jejunum of DIO mice compared to CTRL ones (Figure 1.F), suggesting a reduced number of mitochondria. This was confirmed by mitochondrial observations using transmission electron microscopy that showed a reduction by half of the number of mitochondria in IEC from DIO compared to CTRL mice (Figure 1.G). Moreover, mitochondria appeared elongated in IEC from DIO mice (CTRL: 0.86 ± 0.02 vs DIO: 0.97 ± 0.02 μm, *P* < 0.001). mRNA relative expression of *Pgc1a*, the master gene regulator of mitochondrial biogenesis, was 60% lower in IEC from DIO compared to CTRL mice (Figure 1.H), with no change in the gene expression of the downstream mitochondrial biogenesis regulators (Figure 1.H). Moreover, gene expression of proteins involved in mitochondrial fusion and fission and in mitophagy was lower in IEC from DIO mice compared to CTRL ones (Figure 1.H).

### 3.3 DIO induces enterocyte steatosis and reduces FAO capacity of mouse jejunal epithelial cells

**Figure 2:**
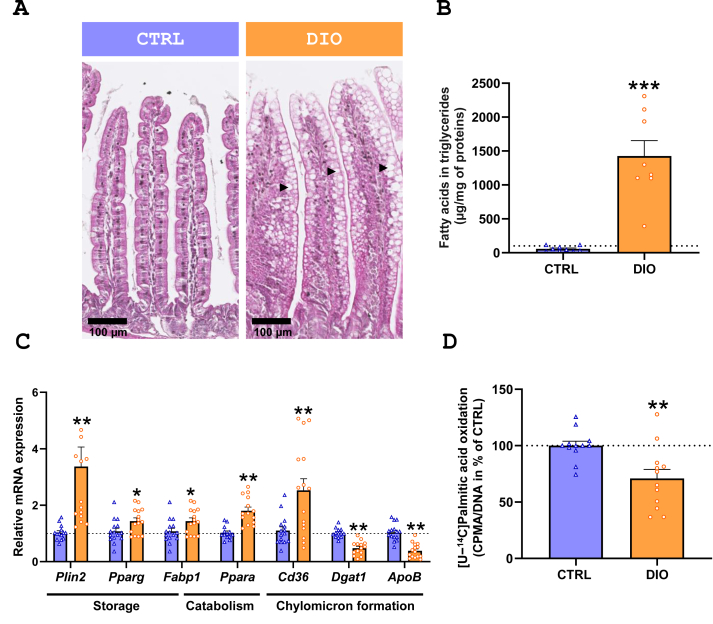
**Diet-induced obesity favours lipid storage and diminishes β-oxidative capacity in IEC. A.** Haematoxylin eosin saffron staining of tip villus from jejunal mucosa of CTRL and diet-induced obesity (DIO) mice. Black arrows indicate lipid droplets in intestinal epithelial cells (IEC). **B.** Quantification of fatty acids from triglycerides of IEC from CTRL and DIO mice (*N* = 9 CTRL; *N* = 8 DIO). **C.** mRNA relative expression of key genes involved in fatty acid metabolism in IEC. Relative gene expressions are presented as fold change relative to CTRL calculated by the 2^−ΔΔCt^ method (*N* = 15 CTRL; *N* = 15 DIO). **D.** Rates of [U–^14^C]palmitic acid oxidation of IEC. Results were normalized by Hoechst intensity and expressed as percentage of CTRL (*N* = 12 CTRL; *N* = 12 DIO). Values are represented as means ± SEM. Significant results are represented with ∗*P* < 0.05, ∗∗*P* < 0.01 and ∗∗∗*P* < 0.001.

Cytosolic lipid droplets were apparent in IEC from the jejunal mucosa of DIO mice on hematoxylin-eosin-saffron stained jejunal section images (Figure 2.A). Lipid accumulation in IEC was confirmed by quantification of fatty acids from triglycerides in isolated IEC (Figure 2.B). In the same line, expressions of *Plin2*, encoding a protein of lipid droplets and *Pparg* were increased in DIO compared to CTRL mice (Figure 2.C). Moreover, concentration of free fatty acids in IEC from DIO mice was almost 3.5 times higher than in CTRL mice (CTRL: 47.6 ± 8.7 vs DIO: 170.4 ± 24.1 μg of fatty acids/mg of proteins, *P* = 0.0001), suggesting saturation of lipid storage in IEC from DIO mice. Furthermore, and although the expression of *Cd36*, involved in chylomicron formation, was more elevated in IEC from DIO mice compared to that of CTRL, that of *Dgat1* and *Apob* was diminished in DIO (Figure 2.C) and suggests impaired chylomicron formation aligning with enhanced lipid storage. Finally, lipid catabolism seemed also altered since expression of *Ppara* and *Fabp1*, notably involved in directing fatty acids towards FAO, were higher in IEC from DIO compared to CTRL (Figure 2.C). Yet, mitochondrial FAO activity, evaluated by measuring oxidation of [U–^14^C] palmitic acid, was lower in IEC of DIO mice compared to CTRL (Figure 2.D).

### 3.4 IEC antioxidant machinery is enhanced by DIO

**Figure 3:**
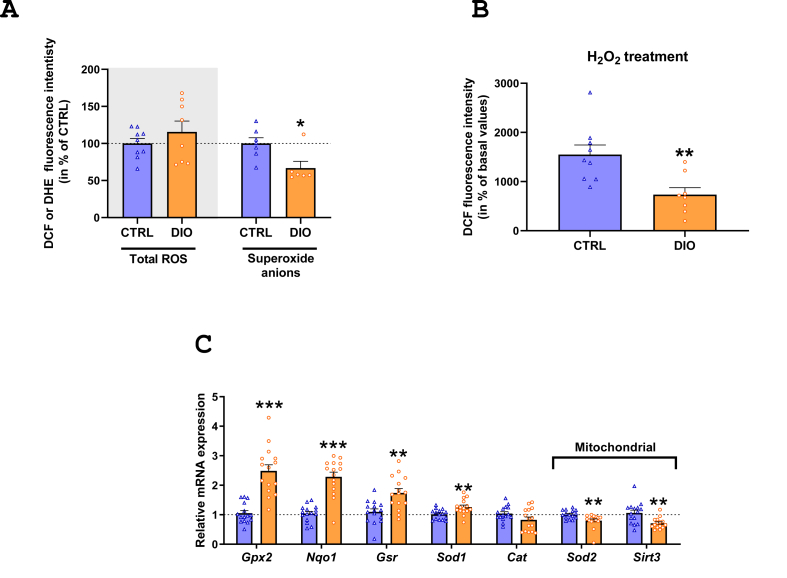
**Diet-induced obesity enhances antioxidant machinery of intestinal epithelial cells. A**. Detection of total reactive oxygen species (ROS) and cytosolic superoxide anions, respectively with H_2_DCFDA and DHE dies, of isolated intestinal epithelial cells (IEC) and normalized per Hoechst fluorescence intensity (*N* = 7–9 CTRL; *N* = 6–8 DIO). Results are expressed in percentage of CTRL. **B.** Detection of total ROS after a 30 min H_2_O_2_ treatment on isolated IEC with the H_2_DCFDA dye. Results are expressed in percentage of CTRL values without H_2_O_2_ treatment. **C.** mRNA relative expression of genes encoding antioxidant enzymes in IEC. Values are presented as fold change relative to CTRL calculated by the 2^−ΔΔCt^ method (*N* = 15 CTRL; *N* = 15 DIO). Results are means ± SEM. Significant results are represented as ∗*P* < 0.05, ∗∗*P* < 0.01, ∗∗∗*P* < 0.001.

Considering the DIO-induced lipid metabolism alterations observed in IEC, we sought to evaluate whether HFD consumption induced oxidative stress in IEC in our model. Peroxide detection in IEC was similar in both groups while superoxide anions were less abundant in IEC from DIO mice compared to CTRL ones (Figure 3.A). Moreover, under external oxidative stress induced by the addition of H_2_O_2_, peroxide increase was 50% lower in IEC from DIO than CTRL mice (Figure 3.B). Consistently, mRNA relative expression of genes involved in the antioxidant machinery (*Gpx2*, *Nqo1*, *Gsr*, *Sod1*) was higher in IEC from DIO than CTRL mice (Figure 3.C). On the contrary, genes that encode the mitochondrial targeted *Sirt3* and *Sod2* were less expressed in IEC from DIO mice than CTRL ones (Figure 3.C).

### 3.5 DIO enhances IEC renewal and reduces mature enterocyte phenotype

**Figure 4:**
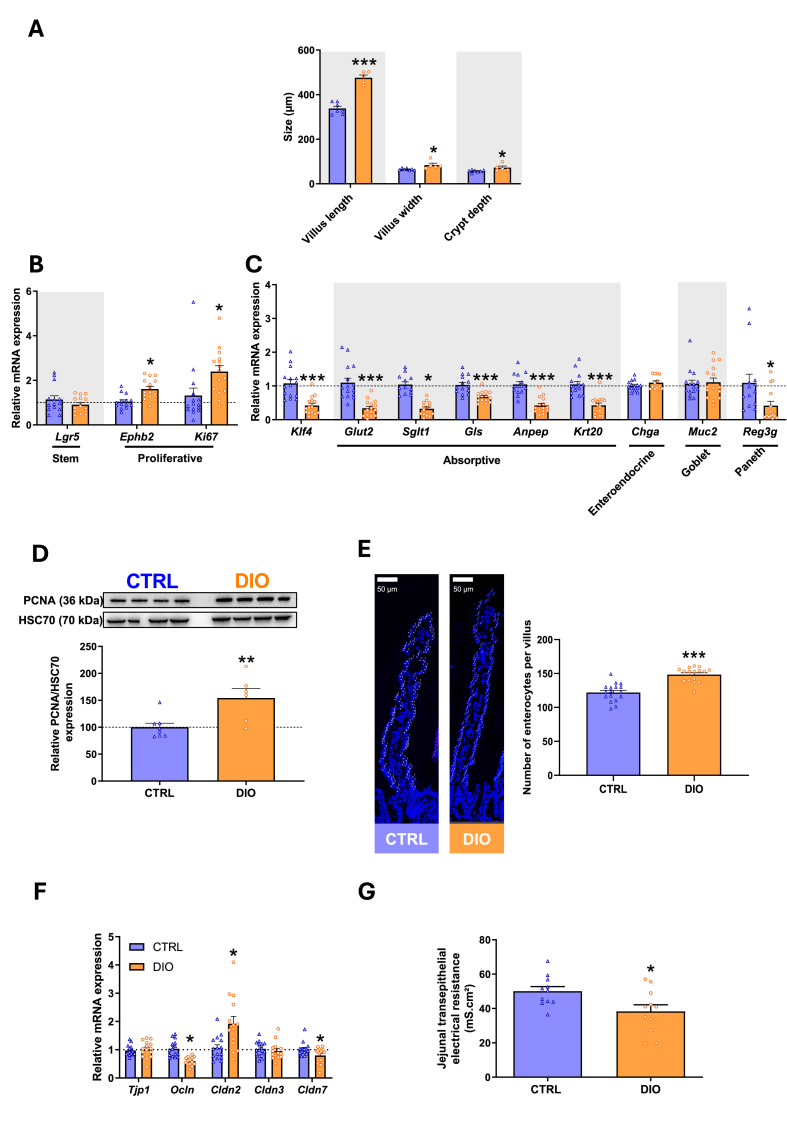
**DIO enhances epithelial cell proliferation, reduces absorptive epithelial cell differentiation in association with increased intestinal permeability. A.** Histological measurement of villus length, width, and crypt depth performed on hematoxylin eosin saffron staining of jejunal mucosa from CTRL and diet-induced obesity (DIO) mice (each dot represents the mean of histological measurements obtained from *N* = 7 CTRL mice and *N* = 5 DIO mice). **B.** mRNA relative expression of stemness and proliferation markers and **C.** of differentiation markers. Values are presented as fold change relative to CTRL calculated by the 2^−ΔΔCt^ method (*N* = 15 CTRL; *N* = 15 DIO). **D.** Protein expression of proliferating cell nuclear antigen (PCNA) in intestinal epithelial cells (IEC) determined by semi-quantitative analysis of western blot reported to Heat shock 70 kDa protein (HSC70) expression. Values are expressed as percentage of CTRL (*N* = 8 CTRL; *N* = 6 DIO). **E.** Number of IEC per villus counted from DAPI (nuclei in blue) staining of jejunal mucosa (each dot represents the number of IEC per villus counted from *N* = 3 CTRL mice and *N* = 3 DIO mice). **F.** Relative mRNA expression of genes encoding tight junction proteins in IEC. Relative mRNA expression is presented as fold change relative to CTRL calculated by the 2^−ΔΔCt^ method (*N* = 15 CTRL; *N* = 15 DIO). **G.** Transepithelial electrical resistance of jejunal mucosa measured in Ussing chamber (*n* = 11 mice per group). Values are expressed as means ± SEM. Significant results are represented as ∗*P* < 0.05, ∗∗*P* < 0.01, ∗∗∗*P* < 0.001. (For interpretation of the references to color in this figure legend, the reader is referred to the Web version of this article.)

Morphometric analysis, performed on hematoxylin-eosin-saffron stained jejunal sections, showed 40% longer and 33% larger villi as well as 26% deeper crypts in the jejunum from DIO mice compared to CTRL ones (Figure 4.A). In line with these data, genes encoding proliferative markers were more expressed (Figure 4.B) whereas that of markers of differentiated absorptive enterocytes and Paneth cells were all decreased by half in IEC from DIO mice compared to CTRL ones (Figure 4.C). Noteworthy, markers of enteroendocrine cells and goblet cells were not altered by DIO, suggesting that DIO altered late differentiation of absorptive cells but not early differentiation of transit amplifying cells into secretive or absorptive lineage. Concomitant to the increase in gene expression of proliferative markers, protein expression of the proliferating cell nuclear antigen (PCNA), involved in DNA replication during cell division, was 50% higher in IEC of DIO mice than that of CTRL (Figure 4.D) and the average number of IEC per villus was higher in DIO mice than CTRL mice (Figure 4.E). In line with this enhanced proliferation/lower differentiation of IEC in DIO mice, the expression of genes encoding for the TJ proteins were altered, with reduced *Cldn7* and *Ocln* and enhanced *Cldn2* expression in DIO compared to CTRL mice (Figure 4.F). In accordance with these TJ protein remodeling, transepithelial electrical resistance was lower in the jejunum of DIO than CTRL mice, indicating increased TJ permeability in jejunum of DIO mice (Figure 4.G).

### 3.6 Reduced mitochondrial bioenergetics associated with enhanced epithelial renewal is linked with triglyceride accumulation in IEC

To determine whether triglyceride accumulation was responsible of reduced mitochondrial bioenergetics and differentiation in the jejunal epithelium of DIO mice, another model of obesity was used. When mice were fed for 12 weeks with a western diet (WD), with lower lipid content that in DIO (DIO: 58% kcal vs WD: 45% kcal derived from fat), they developed obesity marked by increased adiposity and hepatic steatosis but histology of jejunal mucosa did not reveal any triglyceride accumulation. In this obesity model, mitochondrial function of IEC and *Pgc1a* expression were not modified by WD consumption compared to chow fed mice. Furthermore, the expression of genes encoding TJ proteins, including *Cldn2*, were unaltered in IEC from WD mice and the expression of proliferative markers was diminished in WD mice compared to chow fed mice (Supplementary Fig. 5).

### 3.7 Changes in lipid metabolism precede mitochondrial alterations and increased intestinal permeability in an *in vitro* model of enterocytes

**Figure 5:**
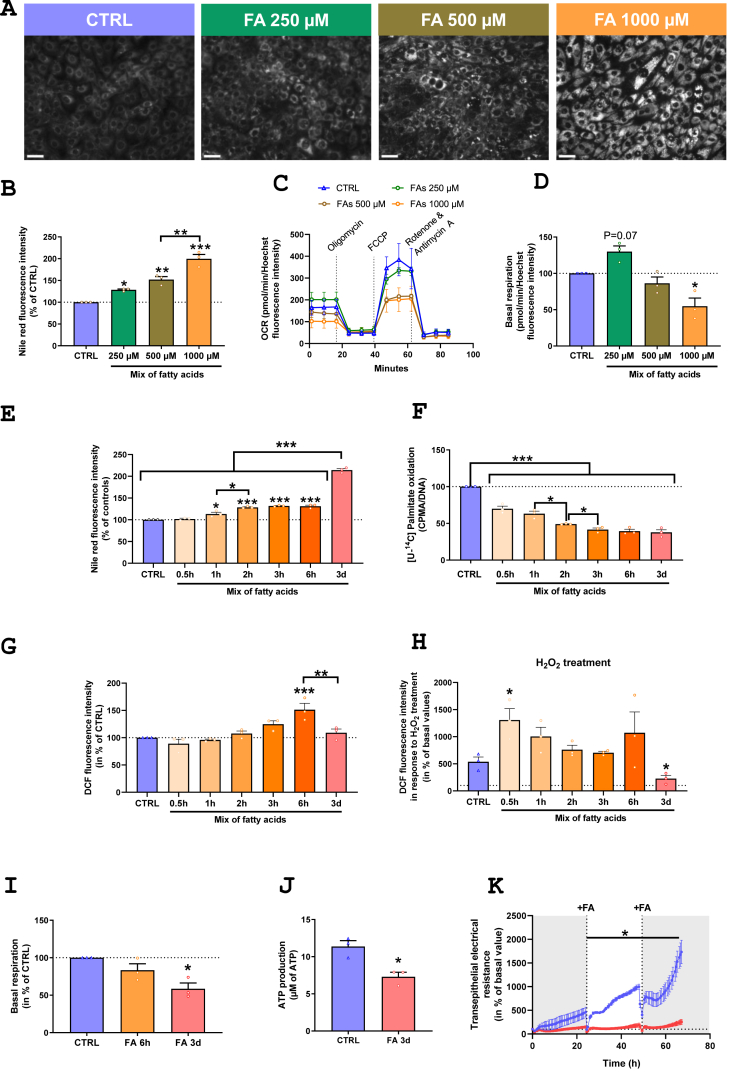
**Fatty acids induce rapid lipid storage into enterocyte before initiating reduced mitochondrial respiration and increased epithelial permeability *in vitro*. A**. Nile red staining and **B.** evaluation of lipid accumulation by quantification of Nile red fluorescence intensity in IPEC-J2 after 3 days of treatment with different concentrations of an equimolar mix of C12:0, C14:0, C16:0 and C18:0. Scale bar represents 50 μm. Data are represented in percentage of CTRL. **C.** Oxygen consumption rate (OCR) profile measured by Seahorse and **D.** basal mitochondrial respiration of IPEC-J2 cells after 3 days of treatment expressed in percentage of CTRL. **E.** Nile red fluorescence intensity of IPEC-J2 cells treated from 0.5 h to 3 days with the mix of fatty acids at 1 mM. Data are represented in percentage of CTRL. **F.** β-oxidation of [U–^14^C]palmitic acid after cells were treated from 0.5 h to 3 days with the mix of fatty acids at 1 mM. Data are expressed in percentage of CTRL. **G.** Basal reactive oxygen species (ROS) detection, represented in percentage of CTRL, and **H.** after H_2_O_2_ treatment, expressed in percentage of basal values, detected by the H_2_DCFDA dye after cells were treated from 0.5 h to 3 days with the mix of fatty acids at 1 mM. **I.** Basal mitochondrial respiration, determined by Seahorse, of IPEC-J2 treated for 6 h or 3 days of the fatty acid mix at 1 mM and expressed in percentage of CTRL**. J.** Measurement of ATP production after a 3-daytreatment with the mix of fatty acids at 1 mM. **K.** Evolution of transepithelial electrical resistance from the onset of fatty acid treatment (Time = 0 h) to 3 days. Fatty acid treatment was renewed every 24 h. Values are expressed as means ± SEM. Significant results are represented as ∗*P* < 0.05, ∗∗*P* < 0.01, ∗∗∗*P* < 0.001 (*N* = 3 different passages for each experiment). (For interpretation of the references to color in this figure legend, the reader is referred to the Web version of this article.)

To determine the sequence of events leading to altered lipid metabolism and reduced mitochondrial function in IEC, we used the IPEC-J2 cell line, an *in vitro* model of jejunal cells to study IEC impacts of lipid overload. Our model of DIO included HFD consumption together with sugars in drinking water. Hence, in order to mimick the effect of DIO *in vitro*, we first evaluated the respective roles of enhanced dietary lipid consumption or of the sugar-supplemented drinking water on IEC phenotype of DIO mice. The comparison of IEC lipid metabolism, bioenergetics and gene expression of mice receiving HFD with either plain or sugar-supplemented drinking water revealed that sugar supplementation in drinking water was not involved in DIO IEC phenotype (Supplementary Fig. 6). Thus, IPEC-J2 cells were treated with an equimolar mix of C12:0, C14:0, C16:0 and C18:0, the main fatty acids found in the obesogenic diet used in this study and in triglycerides of mouse IEC (Supplementary Table 1), to simulate the obesogenic diet. Treatment with the mix of fatty acids for 3 days induced lipid accumulation (Figure 5.A) in IPEC-J2 in a dose-dependent manner from 250 to 1000 μM (Figure 5.B). Yet, mitochondrial function and basal respiration were impaired only when cells were treated with the fatty acid mix at 1 mM (Figure 5.C, D), with a 50% lower basal respiration, similar to that observed in IEC from DIO mice. Accordingly, cell viability evaluated through the MTT test based on mitochondrial function was reduced by a treatment with the mix of fatty acid at 1 mM without significant increased cell death measured through the release of lactate dehydrogenase (Supplementary Fig. 7). To better characterize the succession of events that initiates changes in mitochondrial respiration, IPEC-J2 were next treated with 1 mM of the fatty acid mix, from 0.5 h to 3 days. We first observed that fatty acids were stored in a time dependent manner, starting after 1 h of treatment (Figure 5.E). In the meantime, FAO capacity was reduced with time, starting with a 30% decrease compared to CTRL after 0.5 h of treatment to 60% from 3 h to 3 days (Figure 5.F). ROS production was induced by fatty acid treatment, with a peak of ROS generation at 6 h but a return to basal level at 3 days (Figure 5.G). Moreover, the antioxidant machinery was induced between 6 h and 3 days of fatty acid treatment since the addition of H_2_O_2_ reduced total ROS detection compared to CTRL at 3 days but not 6 h of fatty acid treatment (Figure 5.H). Oxidative stress was even boosted after 0.5 h of fatty acid treatment, as indicated by increased peroxide detection compared to CTRL after H_2_O_2_ treatment at that time point (Figure 5.H). We next evaluated mitochondrial function at different time points and revealed that basal mitochondrial respiration was not significantly altered after 6 h of treatment with fatty acids whereas it was diminished by half after 3 days (Figure 5.I). Consistent with the decrease in basal mitochondrial respiration, ATP concentration was diminished by 33% after a 3-day treatment with the mix of fatty acids compared to CTRL (Figure 5.J). Finally, transepithelial electrical resistance was measured along time from the beginning of the fatty acid treatment. Transepithelial electrical resistance was decreased by fatty acid treatment from 24 h to 3 days compared to CTRL, but not at earlier time points (Figure 5.K).

### 3.8 Energetic status and mitochondrial activity regulate IEC proliferation/differentiation balance

**Figure 6:**
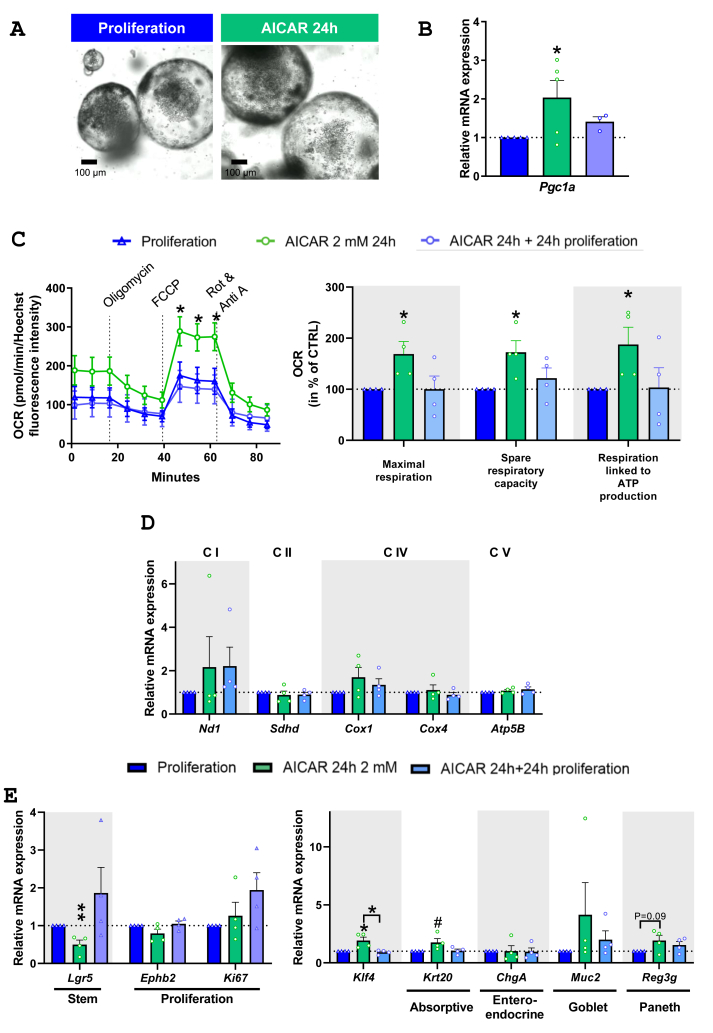
**Changes in energetic status drives the proliferation and differentiation balance of IEC. A**. Images of jejunal organoids in proliferation, treated with 2 mM of AICAR for 24h. **B.** Relative *Pgc1a* expression in mouse jejunal organoids after AICAR treatment for 24h and after AICAR removal for another 24h. **C.** OCR profiles and bioenergetic parameters of jejunal organoids after AICAR treatment for 24h and after AICAR removal for another 24h. **D.** Relative mRNA expression of genes encoding ETC subunits and **E.** proliferating or differentiated intestinal epithelial cell markers in mouse jejunal organoids treated with AICAR. Values are expressed as means ± SEM. Relative mRNA expression is presented as fold change relative to proliferating organoids calculated by the 2^−ΔΔCt^ method. Significant results are represented as ∗*P* < 0.05, ∗∗*P* < 0.01, ∗∗∗*P* < 0.001 and ^**#**^*P* ≤ 0.06 vs proliferating organoids (*N* = 4–5 different organoid lineages).

We next sought to establish the link between IEC energetic status and the proliferation/differentiation balance in the intestinal epithelium using jejunal organoids. We first confirmed that differentiated organoids are characterized by increased *Pgc1a* expression compared to proliferating ones, indicating an association between *Pgc1a* expression and IEC differentiation (Supplementary Fig. 8). Proliferating organoids were treated for 24 h with 2 mM AICAR, an inductor of the AMPK pathway (Figure 6.A). AICAR increased *Pgc1a* expression in organoids maintained in proliferating medium, in a reversible way (Figure 6.B). Accordingly, measures of OCR revealed that AICAR treatment enhanced organoid mitochondrial activity and ATP production, as demonstrated by their increased maximal respiration, spare respiratory capacity and respiration-linked to ATP production compared to untreated organoids (Figure 6.C). This enhanced mitochondrial activity was reversed when removing AICAR (Figure 6.C). Yet, increase in *Pgc1a* expression induced by AICAR was not associated with elevated expression of genes encoding ETC subunits (Figure 6.D). This suggests that enhanced mitochondrial oxidative capacity induced by AICAR was due to increased oxidative activity rather than by elevated mitochondrial biogenesis or expression of ETC complexes. We next used this model of mitochondrial function modulation to explore the link between mitochondrial function and the proliferation/differentiation balance, without change in nutrient supply nor in external proliferative or differentiating factors. Enhancing the energetic status of organoids with AICAR increased the expression of the differentiation markers *Klf4* and *Krt20* and reduced that of the stem cell marked *Lgr5* compared to untreated organoids (Figure 6.E). Removing AICAR for 24 h restored the proliferative phenotype of organoids (Figure 6.E), suggesting that increasing or decreasing mitochondrial function engages IEC towards differentiation or proliferation, respectively.

## 4 Discussion

In this study, we showed that excess dietary lipid consumption reduced mitochondrial number and OXPHOS capacity in mouse jejunal epithelial cells, concomitantly to altered lipid metabolism favoring enterocyte lipid storage. At the same time, IEC renewal was switched towards enhanced proliferation and reduced differentiation and enhanced transepithelial permeability. An *in vitro* model of jejunal epithelial cells allowed us to establish that adaptation of lipid metabolism under lipid overload preceded the reduction in mitochondrial respiration. Finally, using mouse jejunal organoids, we demonstrated that enhancing the IEC mitochondrial function promoted IEC differentiation over proliferation and conversely, suggesting that the mitochondrial adaptations observed in IEC of DIO mice are likely at the origin of the reduced IEC differentiated phenotype and subsequent barrier default.

High fat diet (containing 58% energy from fat) chronic consumption in our study reduced mitochondrial number and OXPHOS capacity in mouse jejunal epithelial cells. Accordingly, a decrease in *Pgc1a* expression has already been described in mouse enterocytes in response to chronic HFD [22,40]. However, the precise mechanisms that could explain this drop of *Pgc1a* expression had not been deciphered so far in the intestine. Our *in vitro* data revealed that diminished mitochondrial respiration and ATP production is likely a downstream effect of lipid metabolism adaptation towards lipid storage within enterocytes. Such a link between enhanced lipid storage and reduced mitochondrial activity has been observed in other tissues, such as the liver [41,42], muscles [43,44] and adipose tissue [27]. Although the precise mechanisms that lead to decreased *Pgc1a* expression is not well documented in DIO, it might be diminished in response to decreased AMPK activity. Indeed, our organoid model clearly establishes that modulation of AMPK activity is directly linked with mitochondrial function. Although reduction of this *Pgc1a* transcriptional activator has been described in a context of high nutrient availability, such as dietary lipids, as shown in liver [45], heart [46] and skeletal muscle [47], AMPK activity in response to HFD remains poorly described in IEC. Only one study showed that the expression of phosphorylated AMPK was diminished in the duodenum of obese patients with type 2 diabetes compared to obese individuals without type 2 diabetes [23]. Hence, we postulate that in response to high energy intake, the energetic status of IEC in DIO mice is altered and provokes reduction of *Pgc1a* expression and mitochondrial activity. Nevertheless, we must acknowledge that deletion of AMPK in mouse IEC fed a HFD had no effect on jejunal permeability [48], suggesting that compensatory mechanisms might be activated in this KO model or that other mechanisms could be at play in our own DIO model. Hence, in our study, Sirt3 gene expression was reduced in IEC from DIO compared to CTRL mice. SIRT3 has been shown to induce *Pgc1a* expression in several tissues, including cardiomyocytes [49] and skeletal muscle [50]. However, Ramachandran et al. showed that IEC specific Sirt3-overexpression does not modify the decrease in *Pgc1a* expression in IEC after HFD consumption [40], thus dismissing the hypothesis of lower *Pgc1a* expression due to altered Sirt3 expression in IEC from DIO mice.

At the basal state, diminished FAO capacity and enhanced lipid storage in the intestine may represent an adaptative response to avoid excessive lipid export and thus systemic hyperlipidemia. Indeed, gavaging HFD-fed mice with olive oil decreased mRNA expression of genes involved in lipolysis and lipid export compared to control mice [51]. This is in line with the diminished expression of *Dgat2* and *ApoB* in the IEC of our DIO mice, both involved in chylomicron formation and secretion. Collectively, our data indicates enhanced lipid storage in enterocytes and decreased FAO and lipid export. Yet, this metabolic adaptation seems to occur only above a certain amount of lipids and/or diet duration. Indeed, acute exposure of lean mice to lipids or HFD (30% kcal from fat) feeding on a short time (2 weeks) resulted in increased expression of genes involved in fatty acid catabolism, triglycerides and chylomicron synthesis as well as elevated FAO capacity [21]. Similarly, when using a diet with lower amount of fat and a shorter period, we did not observe any lipid droplet accumulation nor diminished *Pgc1a* expression or changes in mitochondrial function in DIO mice. Likewise, decrease in basal respiration of IPEC-J2 was induced only in response to high concentration of fatty acids in our study. Recently, Moschandrea et al. established that inducing mitochondrial dysfunction in mouse IEC, through the ablation of genes encoding OXPHOS subunits, resulted in decreased OXPHOS complex activity, increased lipid storage and diminished chylomicron export in the small intestine of mice [38]. Yet, our model revealed that lipid accumulation precedes the decreased of OXPHOS in IEC. Thus, reduced mitochondrial biogenesis and activity in IEC is likely to be induced in response to increased lipid storage in the small intestine.

Intestinal epithelium from DIO mice was marked by increased IEC proliferation and diminished expression of differentiation markers compared to CTRL. Accordingly, recent works showed that HFD consumption augments ISC proliferation and intestinal stemness by activating PPARδ and PPARα [11]. Reduced differentiation of IEC in DIO mice is likely linked to the observed reduced mitochondrial function of IEC. OXPHOS activity that stimulates the ROS-driven p38 mitogen-activated protein kinase activation has been previously shown to be required when reduced Wnt signaling factors-induced crypt formation in mouse small intestine organoids [16]. Here, we demonstrate that enhanced mitochondrial function is not only required but sufficient to commit IEC towards differentiation since stimulating mitochondrial function of intestinal organoids with AICAR changed proliferation and differentiation gene expression, without reducing the growth factors from the proliferating medium. Noteworthy, reducing mitochondrial function recommitted cells towards proliferation, suggesting a highly flexible link between IEC energetic status and their cellular fate. It is thus conceivable that diminished mitochondrial function, associated with high induction of antioxidant machinery in IEC from DIO mice that would prevent the induction of the ROS-driven p38 signaling pathway, impaired IEC differentiation in our study.

Consistent with the increased number of undifferentiated IEC in DIO mice, TJ protein expression differed between IEC from CTRL and DIO mice. Similarly, others showed that HFD induces TJ restructuring and lower expression of genes encoding non-permissive Cldn while *Cldn2* expression was increased. Noteworthy, the enhanced *Cldn2* expression likely participated to the lower transepithelial electrical resistance observed in the jejunum from DIO mice in our study [52]. Fatty acids are known to alter intestinal permeability through different mechanisms such as TJ disruption (protein localization or expression), alterations in the mucus layer or oxidative stress induction as reviewed in [53]. Our group recently showed that individual saturated fatty acids induces mitochondrial dysfunction and alterations of lipid metabolism that lead to increased epithelial permeability *in vitro* in IPEC-J2 [26]. Likewise, we showed here that a 3-day treatment with a mix of fatty acids decreased IPEC-J2 mitochondrial basal respiration and ATP production in association with increased paracellular permeability. Accordingly, it is well known that pharmacological uncoupling of OXPHOS or *in vitro* mitochondrial complex inhibition resulted in decreased ATP production and alterations of TJ [54,55]. Hence, we suggest that the effect of excess dietary lipid on epithelial barrier function is not only linked to the reduced energy provision to maintain TJ but also to an indirect effect on the proliferation/differentiation balance inducing TJ remodeling favoring increased permeability.

## 5 Conclusions

In conclusion, we showed that excessive consumption of lipids induces a decrease in mitochondrial number, respiration, and FAO capacity in IEC from obese mice. The diminished mitochondrial activity participates to the loss of epithelial homeostasis by favoring enhanced proliferation and immature phenotype at the expense of differentiation into mature IEC. Immature intestinal phenotype thus participates in the increase in intestinal permeability that characterizes obesity and aggravates metabolic syndrome.

## Funding

TG was partly funded by Region Bretagne (grant no.300000592).

## CRediT authorship contribution statement

**Thomas Guerbette:** Conceptualization, Data curation, Formal analysis, Investigation, Methodology, Resources, Software, Validation, Visualization, Writing – original draft, Writing – review & editing. **Vincent Ciesielski:** Data curation, Formal analysis, Investigation, Methodology. **Manon Brien:** Investigation, Methodology. **Daniel Catheline:** Formal analysis, Investigation, Methodology. **Roselyne Viel:** Investigation, Methodology. **Mégane Bostoën:** Investigation, Methodology. **Jean-Baptiste Perrin:** Investigation. **Agnès Burel:** Investigation, Methodology, Resources. **Régis Janvier:** Investigation. **Vincent Rioux:** Funding acquisition, Investigation, Methodology, Resources, Writing – review & editing. **Annaïg Lan:** Conceptualization, Funding acquisition, Investigation, Methodology, Project administration, Supervision, Validation, Writing – original draft, Writing – review & editing. **Gaëlle Boudry:** Conceptualization, Data curation, Formal analysis, Funding acquisition, Investigation, Methodology, Project administration, Resources, Supervision, Validation, Visualization, Writing – original draft, Writing – review & editing.

## Declaration of competing interest

The authors declare that the research was conducted in the absence of any conflict of interest.

## Data Availability

Data will be made available on request.
