# Supplementary material for: Bioenergetic adaptations of small intestinal epithelial cells reduce cell differentiation enhancing intestinal permeability in obese mice
Source: Mol Metab. 2025 Jan 13;92:102098. doi: 10.1016/j.molmet.2025.102098 (PMC11795564; doi:10.1016/j.molmet.2025.102098)
Supplement: Multimedia component 1 [file mmc1.docx]

**Supplementary material for**

# **Bioenergetic adaptations of small intestinal epithelial cells reduce cell differentiation enhancing intestinal permeability in obese mice**

Thomas Guerbette, Vincent Ciesielski, Manon Brien, Daniel Catheline, Roselyne Viel, Mégane Bostoën, Jean-Baptiste Perrin, Agnès Burel, Régis Janvier, Vincent Rioux, Annaïg Lan, Gaëlle Boudry

**Supplemental materiel and methods**

**Animal experiments**

To determine the effect of sugar supplementation in drinking water, C57BL/6J male mice received *ad libitum* a CTRL diet (N=9) or an obesogenic diet with tap water (diet-induced obesity (DIO) with tap water, N=9) or with water supplemented with carbohydrates similar to the DIO group from this study (DIO, 45% sucrose and 55% fructose at a concentration of 42 g/L n=9). Euthanasia of mice, tissue collection and IEC isolation were performed as described in *Material and methods*.

Effect of lipid content of the diet on IEC metabolism was determined on 4-week-old C57BL/6N male mice (N=24), from Envigo (Gannat, France) which were housed by group of 4 per cage at 20°C with a 12:12 h light-dark cycle. After a week with a chow diet (Envigo, Teklad Global 16% Protein Rodent Diet), mice received *ad libitum* whether the chow diet (Chow, 5% kcal from fat, N=12) or a western diet (WD, D12451, 45% kcal from fat, N=12). Euthanasia of mice, tissue collection and IEC isolation were performed as described in *Material and methods*.

**Histology**

Morphometric analysis of jejunal mucosa and hepatic observations were performed on microscopic hematoxylin-eosin-saffron stained sections performed at H2P2 platform (Rennes University). Jejunal villus and crypt sizes were measured using the software NDP.view2 (Hamamatsu) and were performed on 3 mice per group (N=5-28 villi or crypts per mouse).

**Hepatic triglyceride quantification**

Hepatic triglyceride quantification was performed as previously described using the DiaSys kit (Diagnostic System, Grabels, France) [1].

**Oral glucose tolerance test**

Oral glucose tolerance test was performed one week before euthanasia. After a 4 h-fast, mouse glycemia was evaluated by collecting blood from tail vein and measuring glycemia with a glucometer. Mice then received a glucose solution (1g/kg) by intra-gastric gavage. Glycemia was measured every 15 min for 1 h and then 90 min and 120 min after oral gavage.

**Bioenergetic analysis**

Extracellular flux analysis were performed with the extracellular flux analyzer Seahorse XFe24 (Agilent Seahorse XF technology). For mouse IEC bioenergetic analysis, IEC suspensions were centrifugated at 200xg for 5 min and resuspended in 200 µL of a mix made of 1:4 (vol:vol) Matrigel diluted in Seahorse complete medium (Agilent, 102353-100, supplemented with 1 mM pyruvate, 2 mM L-glutamine and 17.5 mM glucose). Fifty µL of IEC suspension were seeded per well of the Seahorse plate (100777-004, Agilent) and incubated for 30 min at 37°C in a non-CO_2_ incubator. Seahorse complete medium was added to each well and the plate was incubated for another 15 min at 37°C in a non-CO_2_ incubator. Following incubation, mitochondrial function analysis was performed using the Cell Mito Stress Test kit (Agilent, 103015-100). The bioenergetic analyses of organoids were performed by collecting organoids as previously described and seeding them onto a Seahorse plate pre-coated with Matrigel (1:10 in Seahorse medium). For IPEC-J2, cells were incubated for 45 min at 37 °C in incubator without CO_2_ while the analyzer was calibrating. The Cell Mito Stress Test consisted in injections of oligomycin (2 µM), Carbonyl cyanide-4-phenylhydrazone (FCCP, 2 µM) and antimycin A and rotenone (0.5 µM). Once the assay performed, Seahorse medium was removed and IEC were fixed in 4% paraformaldehyde solution for 30 min and then incubated 15 min with Hoechst 33258 (Thermo Fisher Scientific, H3569), which stains DNA, for normalization. ATP production rate from OXPHOS was determined according to Agilent instructions in the Seahorse Real-Time ATP Rate Assay instructions [2].

**Gene expression**

Total RNA from isolated IEC, organoids and IPEC-J2 were extracted following the manufacturer protocol of the Nucleospin RNA Plus extraction kit (Macherey-Nagel, 740955.250) and assayed using a Spectrometer ND-1000 (Nanodrop). Reverse transcription step was performed using the High-Capacity cDNA Reverse Transcriptase (4368814, Thermo Fisher Scientific). Gene expression was performed by real time quantitative PCR using the PowerSYBR green PCR Master Mix kit (Thermo Fisher Scientific, 4368813). The expression of the genes of interest was analyzed using the 2^-ΔΔCt^ method with Hypoxanthine Phospho Ribosyl Transferase 1 (*Hprt1*) as housekeeping gene. Primer sequences are available on demand.

**Fatty acid quantification from IEC**

IEC were sonicated in 1 mL of phosphate buffer saline (PBS) and 100 µL were kept for protein normalization. 200 µg of C17:0 was added to each sample as an internal standard for quantification. Total lipid extraction was performed as described in [3]. To separate lipid classes, the solution of total lipids was deposited on a silica plate to allow lipid class thin layer chromatography. After migration and revelation with primulin under UV-light, triglycerides and free fatty acids were collected and saponified, with 2 mL NaOH for 15 min at 70°C, and methylated, with 1 mL BF_3_ in methanol for 10 min at 70°C. Five mL of NaCl 0.9% and 300 µL of hexane were added in each tube. After 15 min agitation at room temperature and centrifugation at 1000xg for 10 min, the upper phase was transferred into a new vial. Each sample was injected in split mode (ratio 2:1) in an Agilent 7890N gas chromatograph coupled to a 5975C mass spectrometry detector (Agilent) [4]. The quantified fatty acids were normalized by mg of proteins using the BCA protein assay kit (23225, Thermo Fisher Scientific).

**Detection of reactive oxygen species**

ROS were detected using the H_2_DCFDA (Thermo Fisher Scientific, D399), for total ROS, and dihydroethidium (DHE; Thermo Fisher Scientific, D11347), for cytosolic superoxide anions, dyes. For each animal, total ROS detection was performed at both basal and stressed states. Thus, each IEC suspension was divided in two parts and IEC were centrifugated at 300xg for 5 min and resuspended in a 10 µM DHE solution or in 10 µM H_2_DCFDA ± 33 mM hydrogen peroxide (H_2_O_2_; Sigma Aldrich, 216763) to induce oxidative stress. All reagents were resuspended in HBSS. IEC were incubated with fluorescent dyes for 30 min at 37°C and protected from light. Then IEC were centrifugated at 300xg for 5 min and resuspended in HBSS. Measures of DCFDA fluorescence intensity at ex485/em590 and DHE at ex520/em590 using the POLARstar Omega microplate reader (BMG Labtech) allowed ROS detection. Results were normalized after Hoechst staining and fluorescence intensity measurement.

For IPEC-J2, total ROS were either detected at basal state of after a H_2_O_2_ (3 mM) treatment for 1 h. Cells were then rinsed with HBSS before incubation with H_2_DCFDA probe for 30 min, washed with HBSS and fluorescence was measured. Data were normalized by Hoechst staining.

**IEC FAO capacity assay**

Isolated IEC were centrifugated at 300xg for 5 min and resuspended in 1:4 (vol:vol) Matrigel diluted in William’s medium (Gibco, A12176-01). 40 µL of the IEC suspension were seeded per well of a 96 well plate. The plate was incubated at 37°C, 5% CO_2_ for 10 min to allow the Matrigel to polymerize. For IPEC-J2 FAO assay, cells were seeded onto 96 well plate and treated with the mix of fatty acids for 0.5 h, 1 h, 2 h, 3 h, 6h and 3 days. The day of the assay, treatment was removed, and cells were washed with pre-warmed PBS. For both isolated IEC and IPEC-J2, 100 µL of warm FAO medium was then added to each well and consisted in William’s medium containing 1% bovine serum albumin (BSA), 100 µM palmitic acid (Sigma-Aldrich, P5585), 1 mM L-carnitine (Sigma-Aldrich, C0283) and 0.05 μCi/mL [U-^14^C]-palmitic acid (Perkin Elmer, NEC534050UC). The plate was incubated for 3 h in a 37°C, 5% CO_2_ incubator. After incubation, 1 M perchloric acid was added to each well (100 µL) and incubated at 37°C for 5 min to allow palmitic acid precipitation. The content of each well was homogenized by pipetting up-down several times and centrifugated at 2000xg for 10 min. The supernatants containing soluble FAO metabolites were collected and transferred into a tube containing 4 mL of scintillation fluid. A liquid scintillation analyzer (TRI-CARB 4910TR, PerkinElmer) recorded the disintegrations per minute of each sample. Results were normalized by Hoechst staining performed on a plate, seeded in the same conditions than the one with radioactive components.

**Mitochondria observations**

After fixation of the jejunal mucosa in 2.5% glutaraldehyde (five mm^3^ pieces of mucosa), the specimens were rinsed several times wth 0.15 M sodium cacodylate and post-fixed with 1.5% osmium tetroxide for 1 h. After rinsing, the specimens were dehydrated in increasing concentrations of acetone (50, 70, 80, 90 and 100%, vol:vol). The specimens were gradually infiltrated with increasing concentrations of epoxy resin ( from 35-70 % vol:vol in acetone to 70-35% vol:vol ) for a minimum of 3 h per step, then incubated in pure epoxy resin with 2,4,6-Tris (dimethylaminomethyl)phenol (DMP30, Sigma-Aldrich) and finally polymerized in blocks for 24 h at 60°C. Ultra-thin sections of 80 nm were then cut from the blocks using a UCT ultramicrotome (Leica), placed on grids and post-stained with uranyl acetate for 30 min. Grids were examined at 120 kV with a Jeol 1400 transmission electron microscope equipped with a SC 1000 camera (Gatan Orius) at the MRIc platform (SFR BIOSIT).

TOMM20 immunostaining was performed on jejunal sections for immune-histochemistry of mitochondria. Paraffin-embedded tissue was cut at 4 µm and mounted on positively charged slides before being dried at 58°C for 1 h. Staining was performed on the Discovery ULTRA Automated immunohistochemistry stainer using the ROCHE detection kit. Following deparaffination with Discovery wash solution at 75 °C for 8 min, antigen retrieval was performed using Ventana proprietary Tris-based buffer solution pH 8, at 95°C to 100°C for 40 min. Endogen peroxidase was blocked with 3% H_2_O_2_ for 12 min. After rinsing, slides were incubated at 37°C for 32 min with rabbit anti-TOMM20 antibody (ab186735 Abcam; dilution at 1/5000). Signal enhancement was performed using a Goat anti-Rabbit HRP at 37°C for 16 min and DISCOVERY Rhodamine Kit (542-568nm) for 8 min. Nuclei were stained with DAPI.

**Western blot of proteins from IEC**

Isolated IEC were lysed in RIPA buffer with protease inhibitor (Sigma-Aldrich, R0278) and sonicated before protein quantification, using a BCA protein assay kit (ThermoFisher Scientific, 23225), and denaturation in Laemmli buffer. Ten µg of total protein lysates were separated in 4-12% Bis-tris SDS-PAGE gel and transferred onto PVDF membrane. Membranes were saturated for 2 h at room temperature in a solution of 5% BSA in tris-buffered saline containing 0.1% Tween 20 (TBST). Ponceau staining was performed to serve as loading control. Proteins were then immunoblotted overnight (4°C) with a cocktail of mouse primary antibodies against OXPHOS complex protein subunits (Invitrogen, 45-8199), proliferating cell nuclear antigen (PCNA; Abcam, ab29) and heat shock cognate protein 70 (HSC70; Santa-Cruz, sc7298). After 3 washes, blots were incubated in the appropriate secondary antibodies linked to HRP for 2 h at room temperature in a 5% BSA/TBST solution and enhanced chemiluminescence (Thermo Fisher Scientific, 34076) revealed protein bands. Mean grey value of each band was measured using ImageJ.

**Nile red staining of IPEC-J2**

Nile red staining was performed after cells were fixed into a 4% paraformaldehyde solution for 30 min. The Nile red powder (N1142, Thermo Fisher Scientific) was first diluted to 1 mg/mL into DMSO. Cells were incubated for 30 min at room temperature in a 100 µg/mL Nile red solution, diluted in PBS. After incubation, cells were washed with PBS and fluorescence was read at ex-520/em-590 using the POLARstar Omega microplate reader. Results were normalized using Hoechst 33258.

**ATP production**

ATP production was measured by bioluminescence following the manufacturer’s instructions from the luminescent ATP detection kit (ab113849, Abcam) after a 3-day with the mix of fatty acid 1 mM in IPEC-J2.

**Transepithelial electrical resistance**

Trans-epithelial resistance of jejunal mucosa was recorder using a voltage-clamp as already described [5]. IPEC-J2 transepithelial electrical resistance was measured after seeding cells onto plastic inserts (353504, Falcon) with 0.4 μm pores. 24 h after seeding, cells were treated with the mix of fatty acids. Transepithelial electrical resistance was monitored every 30 min for 3 days using the CellZscope+ (Nano Analytics) and the medium was renewed every 24 h.

**IPEC-J2 viability and death**

IPEC-J2 viability after a 3-day treatment with the mix of fatty acids 1 mM was evaluated by the methylthiazolyldiphenyl-tetrazolium bromide (MTT) assay (M5655, Sigma-Aldrich). MTT (0.5 mg/mL) was added in the well and incubated for 3 h at 37°C, 5% CO_2_. IPEC-J2 were lysed in 0.1M isopropanol/HCl and 2% Triton-X100 for 1 h at room temperature. Cell viability was finally evaluated by measuring absorbance at 570 nm using a BMG Labtech microplate reader (POLARstar Omega). IPEC-J2 death was evaluated by measuring the activity of lactate dehydrogenase (LDH) released in the supernatants of cells after treatment by following the manufacturer’s instructions of the Cytotoxicity Detection Kit (11644793001, Sigma-Aldrich).

**Statistical analysis**

Data are represented as mean ± SEM. Shapiro-wilk normality tests were performed on each data set, followed by a parametric test (Unpaired t-test) between CTRL and DIO mice, if both data sets followed a normal distribution, or a non-parametric test (Mann-Whitney test) otherwise. A total of 32 male mice were used, 16 CTRL and 16 DIO mice. Each dot in Figures 1,2,3,4 and 6 represents the mean per animal. The number of mice per batch was calculated on the basis of the variability due to mouse weights and cellular respiration measurements. As the size of the tissues does not allow all the analyses to be carried out from a single sample, experiments were performed on different batches to obtain a sufficient number of tissues. The effect of fatty acid treatment time on IPEC-J2 was determined by a 2-way ANOVA followed by a Sidak multiple comparison test. A P-value <0.05 was considered significant. Statistical analysis and graphs were performed on GraphPad Prism version 8.0.2.

**Supplementary figure 1. Total amount of proteins obtained after IEC isolation from CTRL or DIO mice.** IEC were isolated from a 8 cm segment of jejunal tissue and proteins were quantified in a fraction obtained after the isolation procedure. Data are represented as means ± SEM. Each dot represents the amount of protein obtained from the jejunum of one mouse (N=23 mice per group).

**Supplementary figure 2. Effect of AICAR treatment on the Hoechst fluorescence intensity of jejunal organoids seeded on Seahorse XFe24 plates and on their bioenergetic.** **A.** Hoechst fluorescence intensity was measured on jejunal organoids seeded on a Seahorse XFe24 after the Cell Mito Stress Test was performed.

**Supplementary Figure 3. Obesogenic diet induces systemic metabolic alterations in mice.** A. Mouse body weight evolution along the 22 weeks of control (CTRL) or obesogenic diet (DIO) consumption (N=9 CTRL; N=8 DIO). B. Weight of peritoneal, epididymal and abdominal fat pads expressed in percentage of mouse body weight (N=9 CTRL; N=8 DIO). C. Haematoxylin eosin saffron staining of liver from CTRL and DIO mice. Scale bar represents 500 µm. D. Quantification of hepatic triglycerides (N=14 CTRL; N=15 DIO). E. Serum alanine aminotransferase (ALAT) concentration (N=13 CTRL; N=15 DIO). F. Blood glycemia during oral glucose tolerance test. G. Serum triglyceride and cholesterol concentrations (N=13 CTRL; N=15 DIO). Values are represented as means ± SEM. Significant results are represented with *P<0.05, **P<0.01 and ***P < 0.001.

**Supplementary figure 4.** **DIO provokes a decrease of IEC bioenergetic parameters.** **A.** Bioenergetic parameters of isolated IEC calculated from the OCR profiles. **B.** Extracellular acidification rate (ECAR) of isolated IEC measured after sequential injection of oligomycin, FCCP and rotenone and antimycin A. **C.** Energy map of isolated IEC obtained from normalized OCR and ECAR data. Data are represented as mean ± SEM. Significant differences are represented as *P < 0.05, **P<0.01, ***P < 0.005 vs CTRL (N=9 CTRL; N=11 DIO).

**Supplementary figure 5. HFD with a lower lipid content prevents enterocyte steatosis, changes in mitochondrial function and reduced proliferation in the jejunum in another model of DIO mice.** C57BL6N mice received a chow diet or a western diet (WD) for 12 weeks (see supplemental materiel and methods). **A.** Mouse body weight evolution along the 12 weeks of Chow diet or WD consumption. **B.** Weight of peritoneal, epididymal and abdominal fat pads expressed in percentage of mouse body weight. **C.** Hematoxylin-eosin-saffron staining of liver or **D.** jejunal mucosa. **E.** Oxygen consumption rate (OCR) profiles and **F.** basal mitochondrial respiration of isolated IEC from mouse jejunum. **G.** Relative mRNA expression of mitochondrial biogenesis regulators. **H.** mRNA relative expression of markers of stemness and differentiation in jejunal epithelial cells. Relative gene expressions are presented as fold change relative to Chow calculated by the 2^-ΔΔCt^ method. Values are expressed as means ± SEM. Significant results are represented as *P<0.05, **P<0.001, ***P<0.001 (N=12 for Chow mice and N=9 for WD mice).

**Supplementary figure 6.** **Sugar-supplemented drinking water is not involved in enterocyte steatosis, mitochondrial alterations and increased antioxidant machinery observed in IEC from DIO mice**. **A**. Mouse body weight evolution along the 22 weeks of control diet (CTRL) consumption or obesogenic diet with plain water (DIO with plain water) or water supplemented with fructose and sucrose at 42 g/L (DIO). **B.** Weight of visceral fat pads expressed in percentage of mouse body weight. **C.** mRNA relative expression of *Cd36*, *Plin2* and *Fabp1* of mouse IEC. **D.** Hematoxylin-eosin-saffron staining of jejunal mucosa. **E.** Quantification of fatty acids from triglycerides of IEC. **F.** Oxygen consumption rate (OCR) measurement by Seahorse and **G.** basal respiration. **H.** *Pgc1a* relative expression of mouse IEC. **I.** Detection of total ROS and cytosolic superoxide anions, respectively with H_2_DCFDA and DHE dies, of isolated IEC and normalized per DNA content. Results are expressed in percentage of CTRL. **J.** Detection of total ROS after a 30 min H_2_O_2_ treatment of isolated IEC with the H_2_DCFDA dye labelling. Results are expressed in percentage of values without H_2_O_2_ treatment of peroxide detection. **K.** mRNA expression of genes encoding antioxidant enzymes in IEC. Relative mRNA values are presented as fold change relative to CTRL calculated by the 2^-ΔΔCt^ method. Results are means ± SEM. Significant results are represented as *P<0.05, **P<0.01, ***P<0.001 (N=9 per group).

**Supplementary figure 7. A mix of fatty acids at 1 mM decreases IPEC-J2 viability without significantly increasing cell death *in vitro*.** **A.** MTT assay to determine IPEC-J2 viability and **B.** LDH test to evaluate cell death after a 3-day treatment with an equimolar mix of C12:0, C14:0, C16:0 and C18:0 (250 µM each). Representative images show the staining after MTT or cytotoxicity detection kit incubations. Data are represented as mean ± SEM. Significant differences are represented as **P<0.01 vs CTRL (N=3).

**Supplementary figure 8. Differentiated organoids are characterized by increased gene expression of *Pgc1a* and markers of mature IEC compared to proliferating organoids. A**. Image of jejunal organoids in differentiation medium for 24h. **B.** Relative mRNA expression of genes encoding proliferating or differentiated intestinal epithelial cell markers and of **C.** *Pgc1a*. Values are expressed as means ± SEM. Relative mRNA expression is presented as fold change relative to proliferating organoids calculated by the 2^-ΔΔCt^ method. Significant results are represented as *P<0.05, ***P<0.001 and **^#^**P≤0.06 vs proliferating organoids (N=3-4 different organoid lineages).

**References for the Supplementary material**

[1] Appriou, Z., Nay, K., Pierre, N., Saligaut, D., Lefeuvre-Orfila, L., Martin, B., et al., 2019. Skeletal muscle ceramides do not contribute to physical inactivity-induced insulin resistance. Applied Physiology, Nutrition, and Metabolism 44(11): 1180–8, Doi: 10.1139/apnm-2018-0850.

[2] Lan, A., Guerbette, T., Andriamihaja, M., Magnin, B., Bordet, M., Ferron, P.-J., et al., 2023. Mitochondrial remodeling and energy metabolism adaptations in colonic crypts during spontaneous epithelial repair after colitis induction in mice. Free Radical Biology and Medicine 205: 224–33, Doi: 10.1016/j.freeradbiomed.2023.06.007.

[3] Guerbette, T., Rioux, V., Bostoën, M., Ciesielski, V., Coppens-Exandier, H., Buraud, M., et al., 2024. Saturated fatty acids differently affect mitochondrial function and the intestinal epithelial barrier depending on their chain length in the in vitro model of IPEC-J2 enterocytes. Frontiers in Cell and Developmental Biology 12.

[4] Drouin, G., Catheline, D., Guillocheau, E., Gueret, P., Baudry, C., Le Ruyet, P., et al., 2019. Comparative effects of dietary n-3 docosapentaenoic acid (DPA), DHA and EPA on plasma lipid parameters, oxidative status and fatty acid tissue composition. The Journal of Nutritional Biochemistry 63: 186–96, Doi: 10.1016/j.jnutbio.2018.09.029.

[5] Lessard, M., Boudry, G., Sève, B., Oswald, I.P., Lallès, J.-P., 2009. Intestinal physiology and peptidase activity in male pigs are modulated by consumption of corn culture extracts containing fumonisins. The Journal of Nutrition 139(7): 1303–7, Doi: 10.3945/jn.109.105023.
