# Supplementary material for: Bioenergetic adaptations of small intestinal epithelial cells reduce cell differentiation enhancing intestinal permeability in obese mice
Source: Mol Metab. 2025 Jan 13;92:102098. doi: 10.1016/j.molmet.2025.102098 (PMC11795564; doi:10.1016/j.molmet.2025.102098)
Supplement: Multimedia component 2 [file mmc2.docx]

**Supplementary Table 1. Fatty acid in triglycerides in intestinal epithelial cells from CTRL and DIO mice.** Fatty acid quantity is expressed in µg per mg of proteins. Data are represented as mean ± SEM. Significant differences are indicated by ***P<0.001 vs CTRL after Student's t test.

| Fatty acids | CTRL | DIO | P-VALUE |
| --- | --- | --- | --- |
| C8:0 | 0.42 ± 0.27 | 1.25 ± 2.56 | <0.001 |
| C10:0 | 2.29 ± 1.76 | 12.16 ± 29.49 | <0.001 |
| C12:0 | 22.93 ± 24.09 | 106.41 ± 248.7 | <0.001 |
| C14:0 | 6.78 ± 5.75 | 48.47 ± 124.69 | <0.001 |
| C14:1 n-5 | 0.07 ± 0.04 | 1.07 ± 2.98 | <0.001 |
| C15:0 | 0.36 ± 0.15 | 0.56 ± 0.71 | <0.001 |
| C16:0 | 11.08 ± 5.91 | 36.3 ± 76.20 | <0.001 |
| C16:1 n-9 | 0.28 ± 0.14 | 0.47 ± 0.69 | <0.001 |
| C16:1 n-7 | 0.83 ± 0.48 | 4.43 ± 10.89 | <0.001 |
| C18:0 | 3.70 ± 2.06 | 25.39 ± 65.59 | <0.001 |
| C18:1 Trans | 0.04 ± 0.04 | 0.43 ± 1.16 | <0.001 |
| C18:1 n-9 | 3.79 ± 4.23 | 18.57 ± 44.88 | <0.001 |
| C18:1 n-7 | 0.17 ± 0.07 | 0.96 ± 2.42 | 0.15 |
| C18:2 n-6 | 3.78 ± 2.08 | 3.16 ± 2.10 | 0.15 |
| C18:3 n-3 | 0.11 ± 0.08 | 1.06 ± 2.87 | <0.001 |
| C20:0 | 0.03 ± 0.02 | 0.72 ± 2.08 | <0.001 |
| C20:1 | 0.17 ± 0.15 | 1.43 ± 3.89 | <0.001 |
| C20:3 n-6 | 0 ± 0 | 0.04 ± 0.12 | <0.001 |
| C20:4 n-6 | 0.09 ± 0.07 | 0.29 ± 0.59 | <0.001 |
| C22:6 n-3 | 0.05 ± 0.05 | 0.2 ± 0.46 | 0.01 |
