# Supplementary figures and images for: Bioenergetic adaptations of small intestinal epithelial cells reduce cell differentiation enhancing intestinal permeability in obese mice
Source: Mol Metab. 2025 Jan 13;92:102098. doi: 10.1016/j.molmet.2025.102098 (PMC11795564; doi:10.1016/j.molmet.2025.102098)

## Slide 1
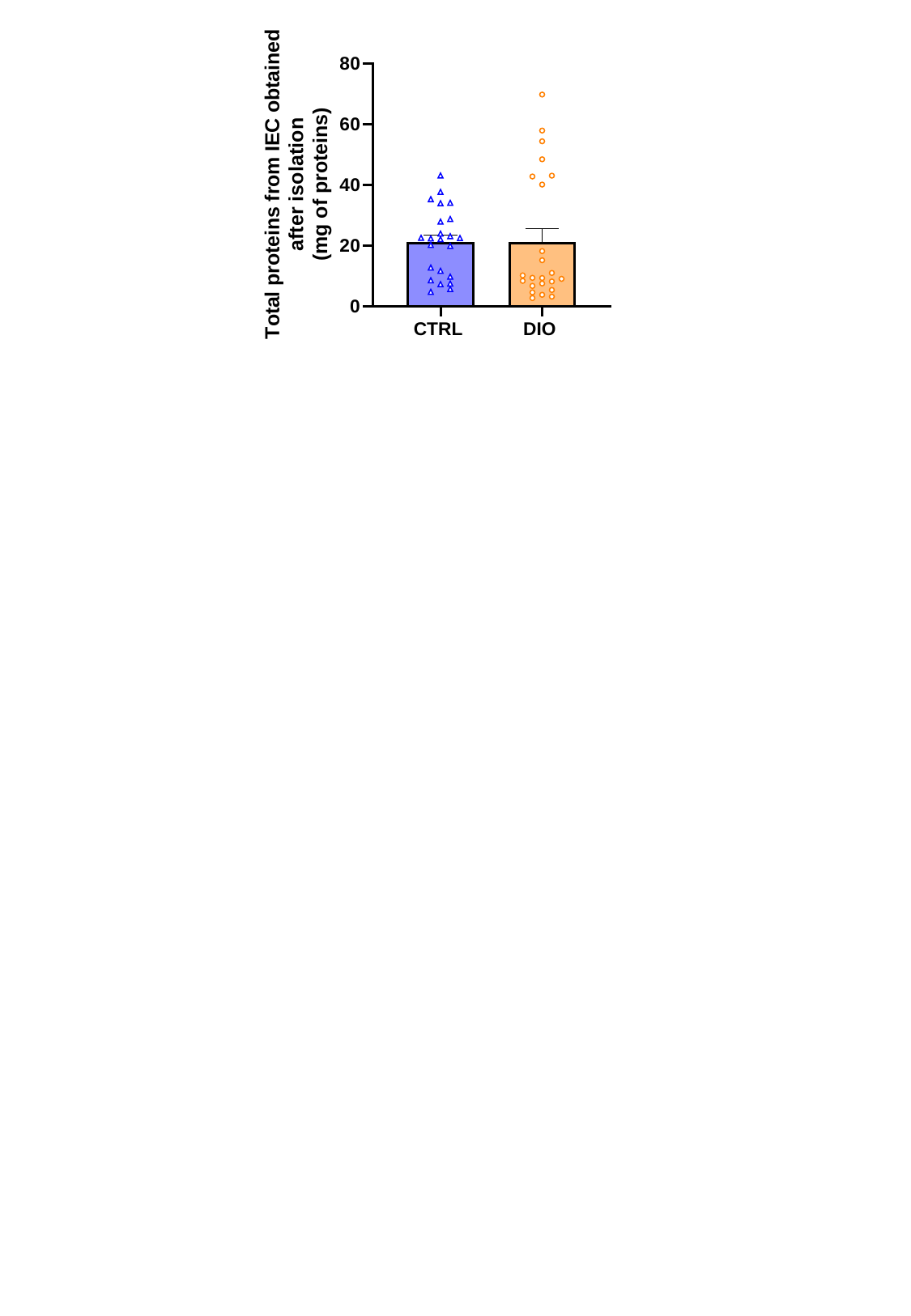

Supplement: Multimedia component 3 [file mmc3.pptx]

## Slide 1
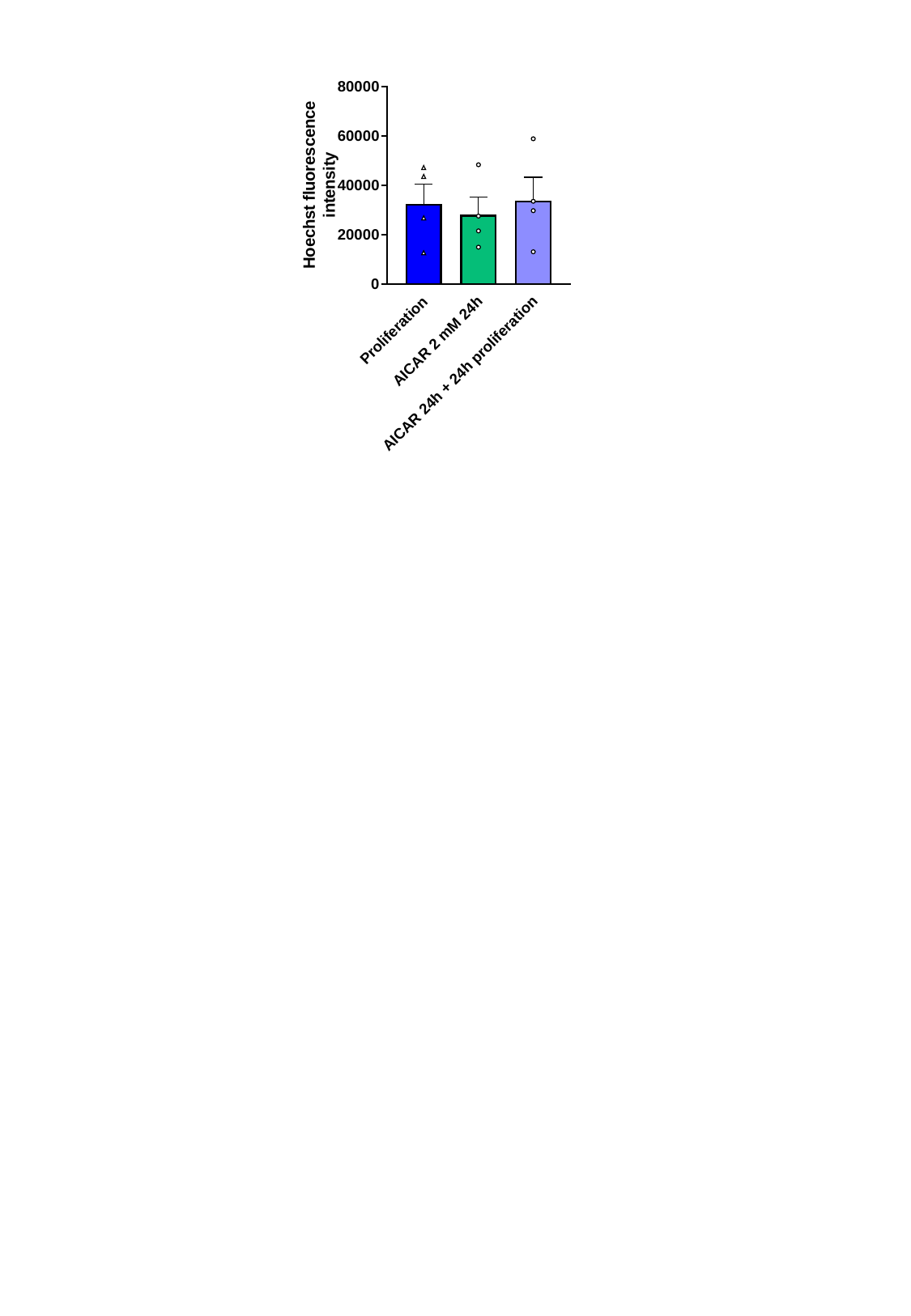

Supplement: Multimedia component 4 [file mmc4.pptx]

## Slide 1
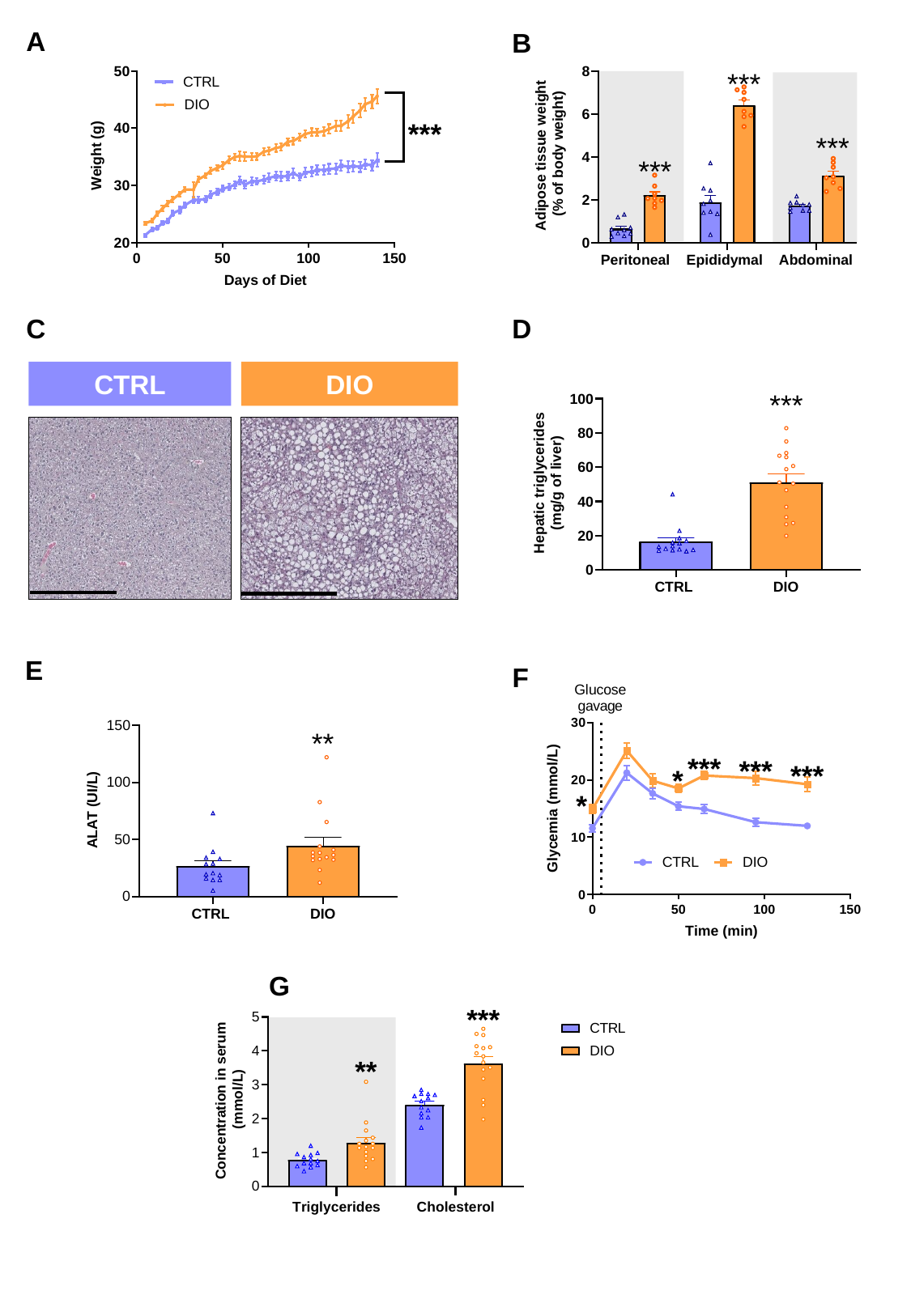

A
B
C
D
CTRL
DIO
E
F
G

Supplement: Multimedia component 5 [file mmc5.pptx]

## Slide 1
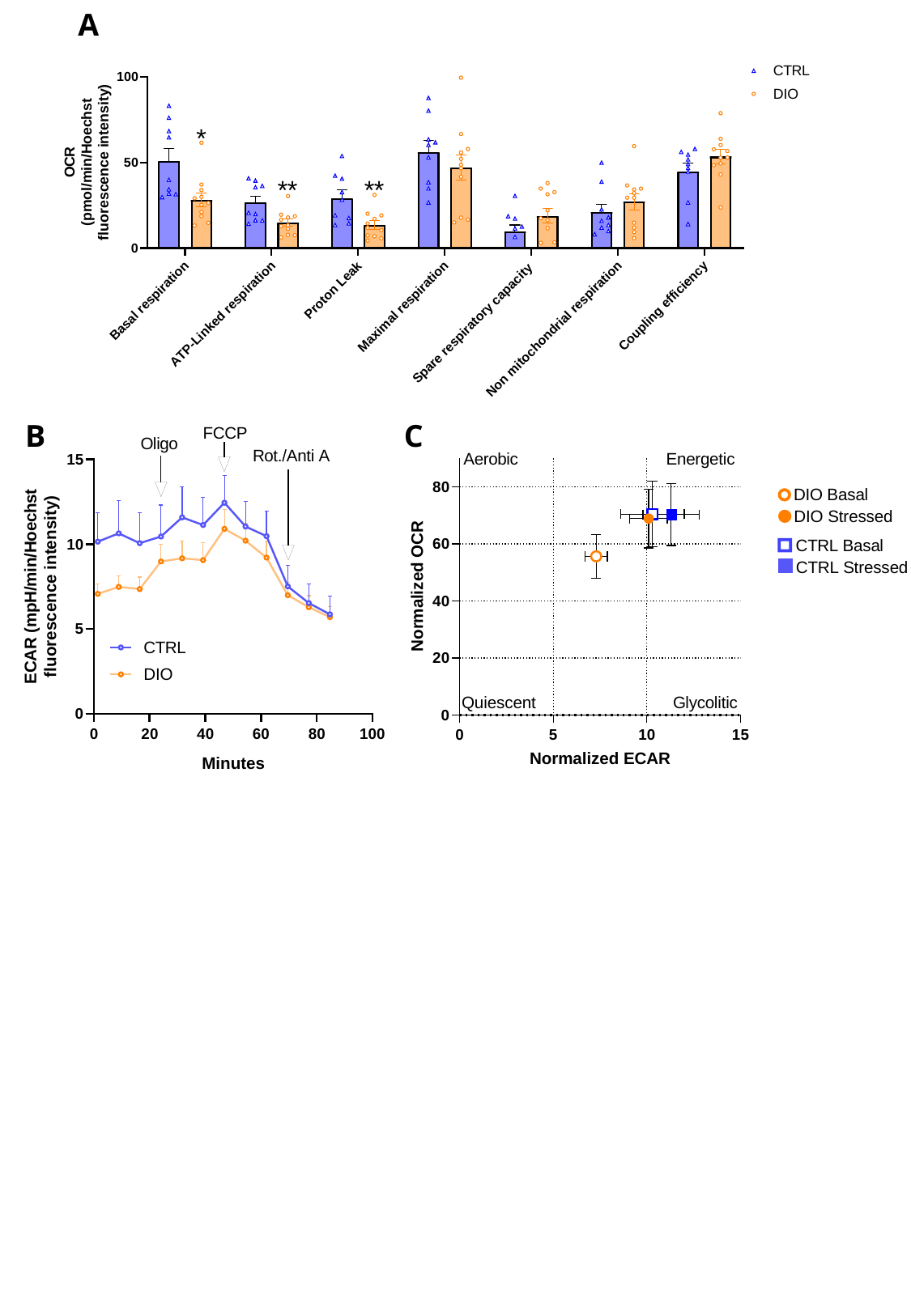

A
B
C

Supplement: Multimedia component 6 [file mmc6.pptx]

## Slide 1
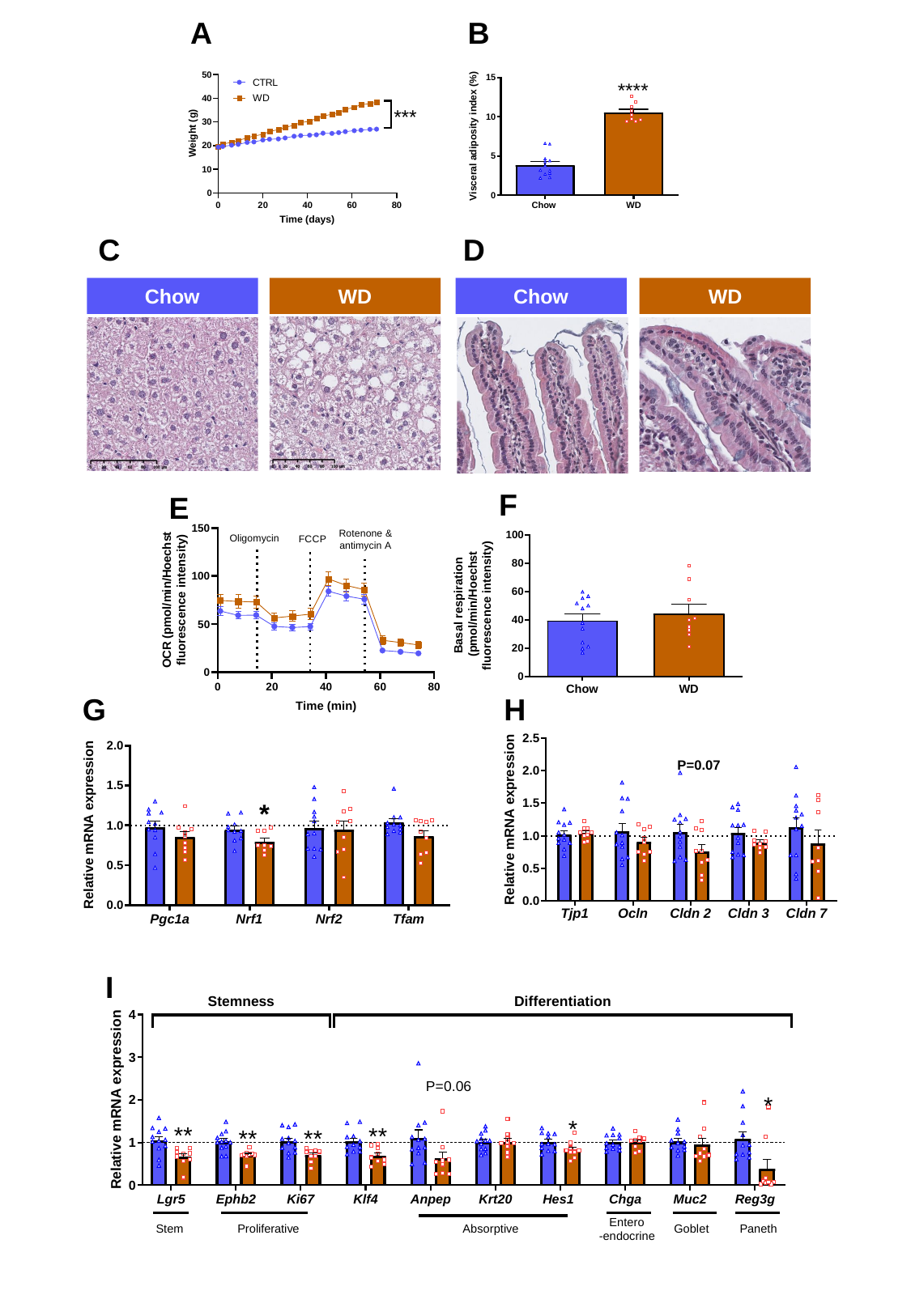

A
B
D
C
Chow
WD
Chow
WD
F
E
G
H
I

Supplement: Multimedia component 7 [file mmc7.pptx]

## Slide 1
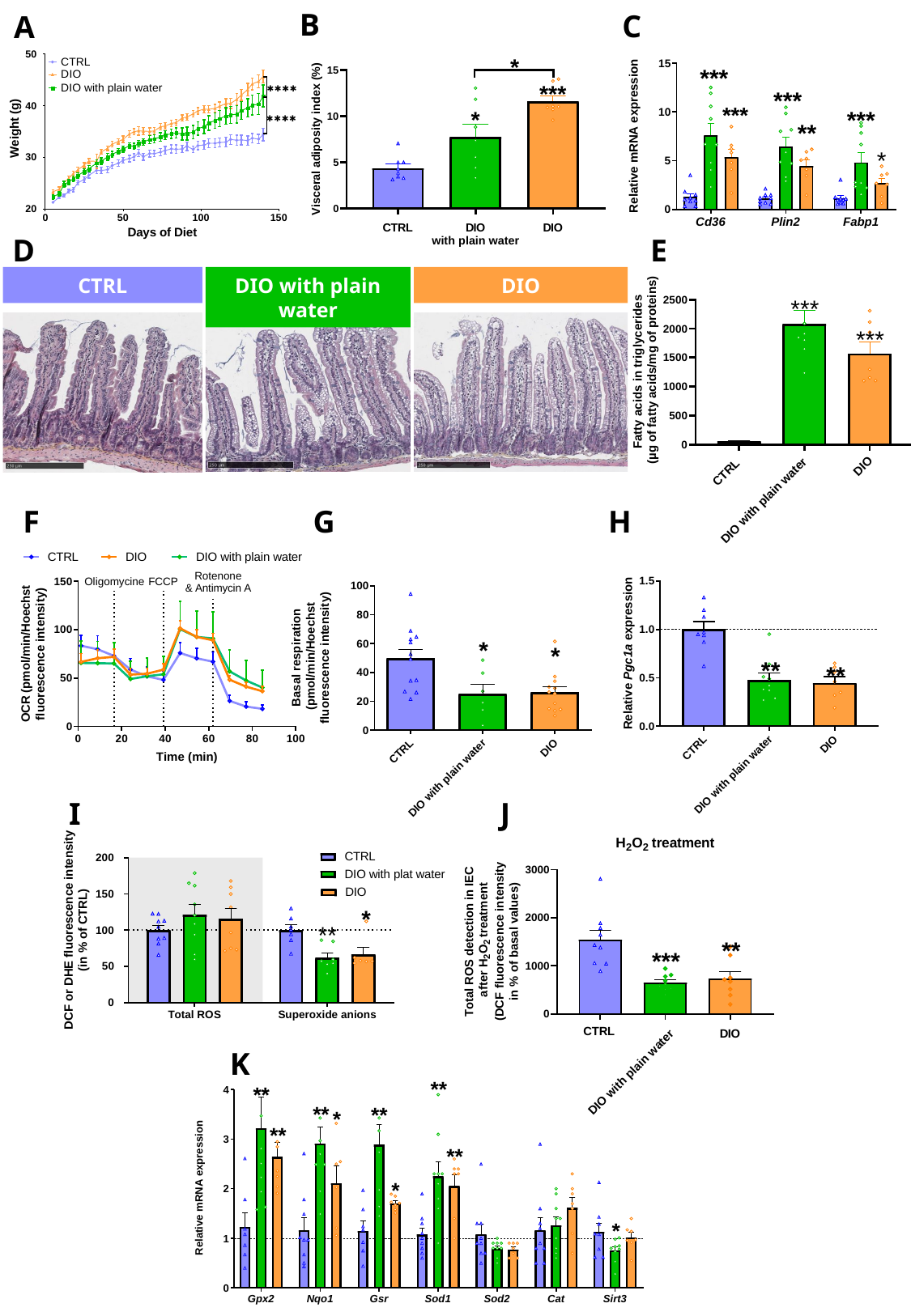

B
C
A
D
E
CTRL
DIO with plain water
DIO
F
G
H
I
J
K

Supplement: Multimedia component 8 [file mmc8.pptx]

## Slide 1
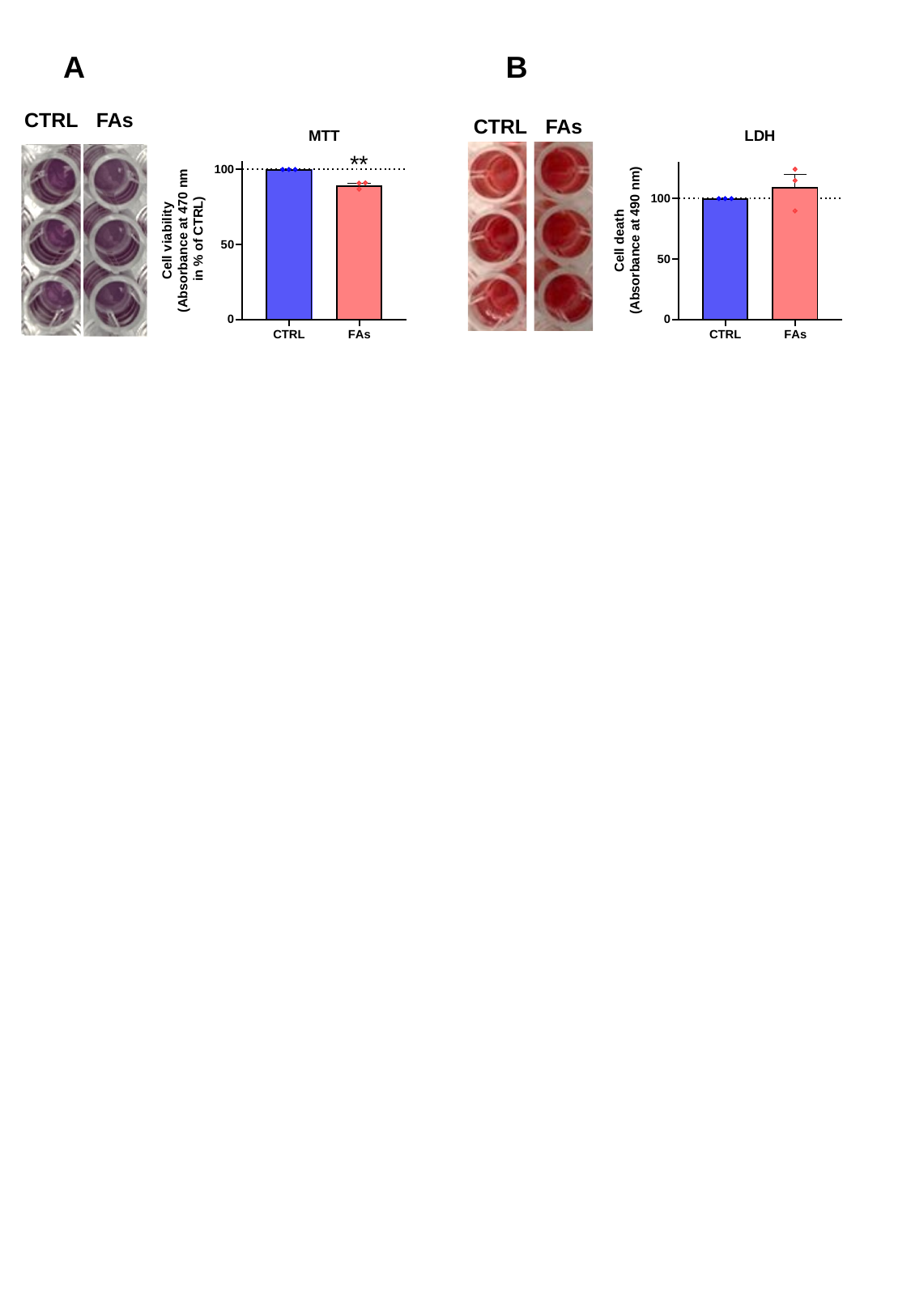

A
B
CTRL
FAs
CTRL
FAs

Supplement: Multimedia component 9 [file mmc9.pptx]

## Slide 1
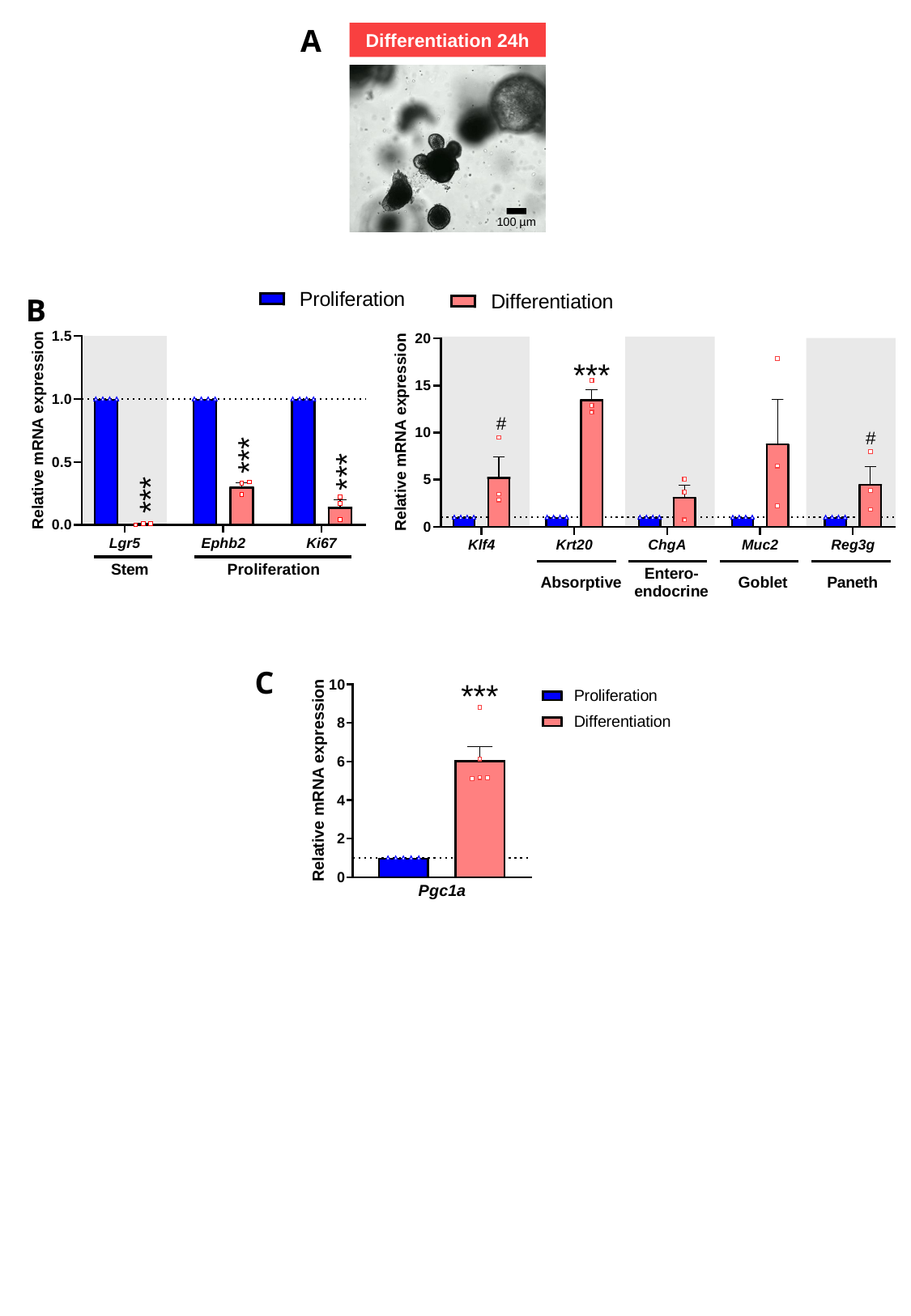

A
Differentiation 24h
100 µm
B
C

Supplement: Multimedia component 10 [file mmc10.pptx]
